# Anti-Mycoplasma Activity of Bacilotetrins C–E, Cyclic Lipodepsipeptides from the Marine-Derived *Bacillus subtilis* and Structure Revision of Bacilotetrins A and B

**DOI:** 10.3390/md19100528

**Published:** 2021-09-22

**Authors:** Hwa-Sun Lee, Hee Jae Shin

**Affiliations:** Marine Natural Products Chemistry Laboratory, Korea Institute of Ocean Science & Technology, 385 Haeyang-ro, Yeongdo-gu, Busan 49111, Korea; hwasunlee@kiost.ac.kr

**Keywords:** *Bacillus subtillus*, cyclic lipodepsipeptide, anti-mycoplasma activity

## Abstract

*Mycoplasma hyorhinis* most commonly causes polyserositis and arthritis in swine and is a common contaminant during the cell culture in the laboratory. In our continuing research for diverse bioactive compounds from *Bacillus subtilis* 109GGC020, we discovered uncommon cyclic lipotetrapeptides showing inhibitory activities against *M. hyorhinis* with similar structures to previously reported bacilotetrins A and B. Bacilotetrins C–E (**1**–**3**), new cyclic lipodepsipeptides, were isolated from the EtOAc extract obtained from the fermentation of marine-derived *Bacillus subtilis* isolated from a marine sponge sample collected from the Gageo reef, Republic of Korea. The structures of **1**–**3**, consisting of three leucine residues, one glutamic acid, and a *β*-hydroxy fatty acid, were elucidated by detailed analysis of 1D, 2D NMR, and HR-ESIMS data. The absolute configurations of the amino acids and *β*-hydroxy fatty acid were established by advanced Marfey’s method and Mosher’s method, respectively. The localization of L- and D-amino acids within the compounds was determined by retention time comparison of each purchased dipeptide standard to the partial hydrolysate products using LC-MS. Compounds **1**–**3** exhibited anti-mycoplasma activity, with an MIC value of 31 μg/mL, twofold stronger than that of the positive control, BioMycoX^®^. Detailed analysis and comparison of the spectroscopic data between bacilotetrins A (**4**) and B (**5**) and **1**–**3** led us to revise the structures of **4** and **5**.

## 1. Introduction

Marine micro-organisms are recognized principally as a significant resource producing new and bioactive compounds [1]. Marine *Bacillus* species produce structurally diverse secondary metabolites, including lipopeptides, polypeptides, macrolides, fatty acids, polyketides, carotenoids, and isocoumarins, which have various activities, such as antimicrobial, anticancer, and antialgal activities [2]. In particular, strong antimicrobial cyclic lipopeptides, including surfactins, iturins, and fengycins, from *Bacillus subtilis* have received great attention for potential biotechnological and pharmaceutical applications [3].

In our previous study, marine-derived *Bacillus subtilis* 109GGC020 has been reported to produce interesting secondary metabolites, including macrolactins (gageomacrolactins [4]), linear lipopeptides (gageotetrins A–C [5], gageopeptides A–D [6], and gageostatins A–C [7]), and cyclic lipopeptides (gageopeptins A and B [8], and bacilotetrins A and B [9]) with antibacterial and antifungal activities. Linear lipopeptides, including gageopeptides A–D and gageotetrin B, exhibited inhibitory effects on the wheat blast fungus *Magnaporthe oryzae Triticum* [10] and the results have revealed the potential of these compounds for agricultural antibiotics.

Mycoplasma is generally known as the smallest bacteria that can survive without oxygen and exists in various forms due to their lack of cell walls [11,12]. Mycoplasma species infect animals, plants, insects, and humans and are often found in research laboratories as contaminants in cell culture [11,13]. Among mycoplasmas, *M. hyorhinis* is a commensal bacterium of the upper respiratory tract of swine and it is a pathogenic mycoplasma species found in piglets [14]. In addition, it has been reported to cause polyserositis [15], arthritis [16], conjunctivitis [17], otitis [18], and cell culture contamination [13].

**Figure 1:**
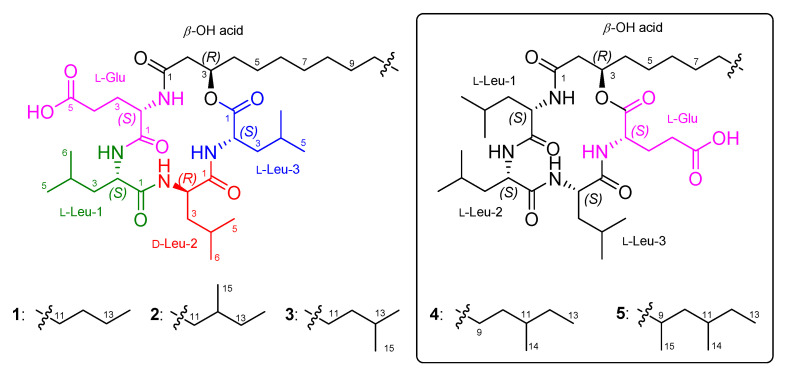
The structures of **1**–**3** and bacilotetrins A (**4**) and B (**5**).

During the investigation for antimicrobial compounds against agricultural pathogens, we discovered new cyclic lipotetradepsipeptides (**1**–**3**) (Figure 1) from the strain 109GGC020 exhibiting inhibitory activities against *Mycoplasma hyorhinis*. The molecular formulae, ^1^H, ^13^C, and 2D NMR data of **1**–**3** were closely similar to previously reported bacilotetrins A (**4**) and B (**5**) [9]. By the detailed and careful analysis of NMR data, we also found that the planar structures of **4** and **5** were wrongly determined. Here, we described the isolation, structure determination, and anti-mycoplasma activity of **1**–**3**, and structure revision of **4** and **5**.

## 2. Results and Discussion

### 2.1. Identification of Isolated Compounds

**Figure 2:**
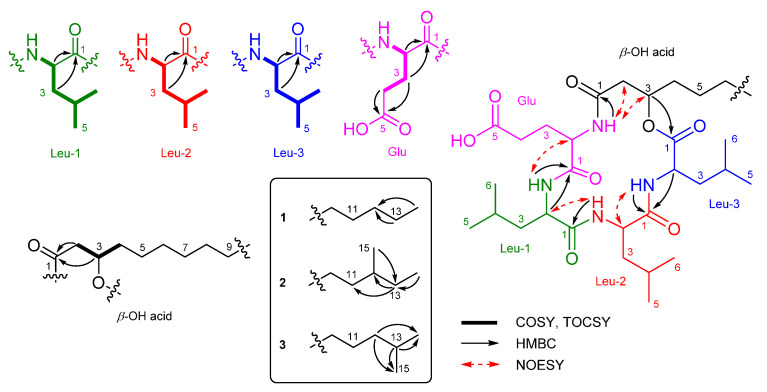
Partial structures and key 2D NMR correlations of **1**–**3**.

Bacilotetrin C (**1**) was obtained as an amorphous solid and its molecular formula was determined to be C_37_H_66_N_4_O_8_ by HR-ESIMS, which required 7 degrees of unsaturation. The NMR data for **1** are summarized in Table 1. The ^1^H NMR (CD_3_OH) spectra suggested the presence of four NH groups (*δ*_H_ 9.10, 8.39, 7.76, and 7.74). ^1^H, ^13^C NMR, and HSQC spectra revealed the presence of four α-protons (*δ*_H_ 4.57, 4.43, 4.11, and 3.74), long aliphatic protons (*δ*_H_ 1.29), one oxygenated proton (*δ*_H_ 5.16), seven methyl protons (*δ*_H_ 0.96–0.89), and six carbonyl carbons (*δ*_C_ 176.3, 175.9, 174.5, 173.9, 173.3, and 172.9). The detailed analyses of ^1^H–^1^H COSY, TOCSY, and HMBC spectra revealed the presence of three leucines (Leu), one glutamic acid (Glu), and a *β*-hydroxy fatty acid (*β*-OH acid). To satisfy the unsaturation number and molecular formula, the structure of **1** was suggested to be a cyclic lipodepsipeptide. The sequence for **1** was determined by analyses of the HMBC and NOESY spectra. The HMBC signals from H-3 (*δ*_H_ 5.16) of *β*-OH acid to C-1 (*δ*_C_ 172.9) of Leu-3, from NH (*δ*_H_ 7.76) and H-2 (*δ*_H_ 4.57) of Leu-3 to C-1 (*δ*_C_ 174.5) of Leu-2, from NH (*δ*_H_ 7.74) of Leu-2 to C-1 (*δ*_C_ 173.9) of Leu-1, from NH (*δ*_H_ 9.10) and H-2 (*δ*_H_ 3.74) of Leu-1 to C-1 (*δ*_C_ 175.9) of Glu, and from NH (*δ*_H_ 8.39) of Glu to C-1 (*δ*_C_ 173.3) of *β*-OH acid established the peptide linkages between the inter-residues. The NOESY correlations between NH (*δ*_H_ 7.76) of Leu-3/H-2 (*δ*_H_ 4.43) of Leu-2, NH (*δ*_H_ 7.74) of Leu-2/H-2 (*δ*_H_ 3.74) of Leu-1, NH (*δ*_H_ 7.74) of Leu-1/H-2 (*δ*_H_ 4.11) of Glu, and NH (*δ*_H_ 8.39) of Glu /H-3 (*δ*_H_ 5.16) of *β*-OH acid also supported the connections between the amino acids and *β*-OH acid. The HMBC signals from the *α*-proton and amide proton of each amino acid to the carbonyl carbon of the adjacent amino acid or *β*-OH acid and the NOESY correlations between the *α*-proton and amide proton of amino acids established the sequence of **1** as (cyclo-(*β*-OH acid-Glu-Leu-1-Leu-2-Leu-3)) (Figure 2). The *β*-OH acid chain was estimated to be a linear type due to the remaining one methyl signal (*δ*_C_ 14.5 and *δ*_H_ 0.89), which matched well with recently reported chemical shifts for the terminal methyl signal (*δ*_C_ 14.4 and *δ*_H_ 0.90) of a linear *β*-OH acid [19].

**Figure 3:**
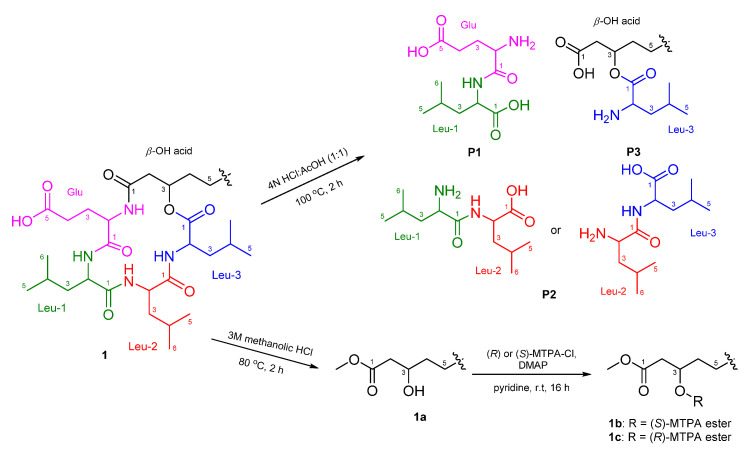
Flow chart for the partial hydrolysis of **1**.

**Figure 4:**
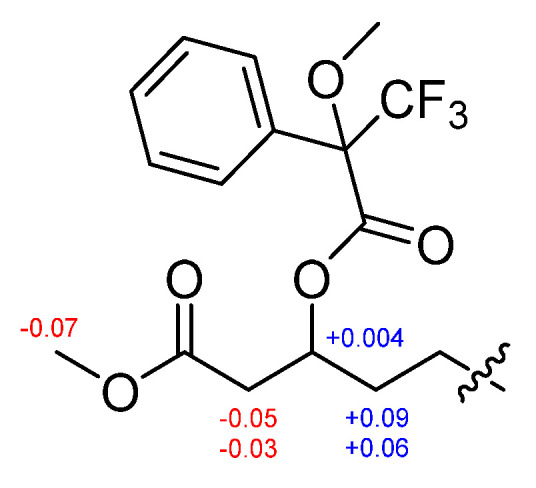
Δ*δ*_H_ = *δ_S_* − *δ_R_* values in ppm for MTPA esters of **1a**.

The absolute configurations of the amino acids in **1** were determined using advanced Marfey’s method [20,21]. The result revealed that **1** contains 2 × L-Leu, 1 × D-Leu, and 1 × L-Glu (Figure S25). To confirm the localization of D-Leu, **1** was subjected to partial hydrolysis, which produced a complex mixture of dipeptides. The mixture was purified by HPLC-MS to give three fragments containing Leu–Glu, Leu–Leu and *β*-OH-acid–Leu (Figure S26). Marfey’s analysis of Leu–Glu and *β*-OH-acid–Leu fragments revealed that these fragments consisted of only the L-form. The Leu–Leu fragment (P2) was reacted with 1-fluoro-2,4-dinitrophenyl-5-L-leucine amide (L-FDLA), which was analyzed by comparing the retention time with L-FDLA derivatives of authentic reagents (L-Leu-D-Leu and D-Leu-L-Leu) (Figure S27). The Leu–Leu fragment (P2) reacted with L-FDLA showed two peaks corresponding with standard derivatives. The retention times of the major peak (*t*_R_ 26.1 min, *m*/*z* 589 [M + H]^+^) and minor peak (*t*_R_ 31.6 min (minor), *m*/*z* 589 [M + H]^+^) of the P2 fragment were in good agreement with those of authentic L-Leu-D-Leu-L-FDLA (*t*_R_ 26.1 min, *m*/*z* 589 [M + H]^+^) and D-Leu-L-Leu-L-FDLA (*t*_R_ 31.6 min, *m*/*z* 589 [M + H]^+^), respectively. Therefore, the Leu–Leu fragment (P2) was a mixture containing Leu-1–Leu-2 (major peak) and Leu-2–Leu-3 (minor peak) fragments. These results indicated that the sequence of three leucines is L–D–L. To determine the absolute stereochemistry of the *β*-OH acid, **1** was cleaved by methanolysis to give a *β*-OH acid methyl ester (**1a**) (Figure 3). Then, **1a** was converted to the (*S*)- (**1b**) and (*R*)-MTPA esters (**1c**) and the ^1^H NMR signals of **1b** and **1c** were designated by the analysis of COSY spectra. The Δ*δ*_H_ values (*δ_S_*_-ester_ − *δ_R_*_-ester_) established the *R* configuration of C-3 in the *β*-OH acid methyl ester (Figure 4).

Bacilotetrins D (**2**) and E (**3**) were isolated as amorphous solids. Both the molecular formulae of **2** and **3** were determined to be C_38_H_68_N_4_O_8_ (unsaturation degree of 7) by HR-ESIMS. The NMR data for **2** and **3** are summarized in Table 1. The ^1^H and ^13^C NMR spectra of **2** and **3** were very similar to those of **1**, except for the branched *β*-OH acids. The main difference in the ^1^H and ^13^C NMR spectra of **2** and **3** lies in the chemical shifts of the terminal methyls in the *β*-OH fatty chains. Bacilotetrin D (**2**) showed anteiso-methyl signals (*δ*_C_ 19.7/*δ*_H_ 0.85 and *δ*_C_ 11.8/*δ*_H_ 0.87), whereas **3** displayed iso-methyl signals (*δ*_C_ 23.1/*δ*_H_ 0.87 × 2). Compound **2** exhibited HMBC signals from H-13 (*δ*_H_ 1.29 and 1.09) of *β*-OH acid to C-11 (*δ*_C_ 30.7) and C-12 (*δ*_C_ 35.7) of *β*-OH acid and from H-14 (*δ*_H_ 0.87) and H-15 (*δ*_H_ 0.85) to C-13 (*δ*_C_ 37.8). These signals established that the branched-chain fatty acid in **2** is an anteiso type. Bacilotetrin E (**3**) also displayed HMBC signals from H-14 and H-15 (*δ*_H_ 0.87) of the *β*-OH acid to C-13 (*δ*_C_ 29.2) of *β*-OH acid and from H-12 (*δ*_H_ 1.16) to C-14 (*δ*_C_ 23.1) and C-15 (*δ*_C_ 23.1) of the *β*-OH acid, suggesting the presence of an iso-methyl branched fatty acid. In addition, anteiso- and iso-methyl signals of **2** and **3** were in good agreement with reference values (anteiso: *δ*_C_ 19.6/*δ*_H_ 0.86 and *δ*_C_ 11.8/*δ*_H_ 0.88; iso: *δ*_C_ 23.1/*δ*_H_ 0.88) [19]. Thus, the sequences of **1**–**3** were identified as cyclo-(*R*-*β*-OH acid-L-Glu-L-Leu-D-Leu-L-Leu).

**Figure 5:**
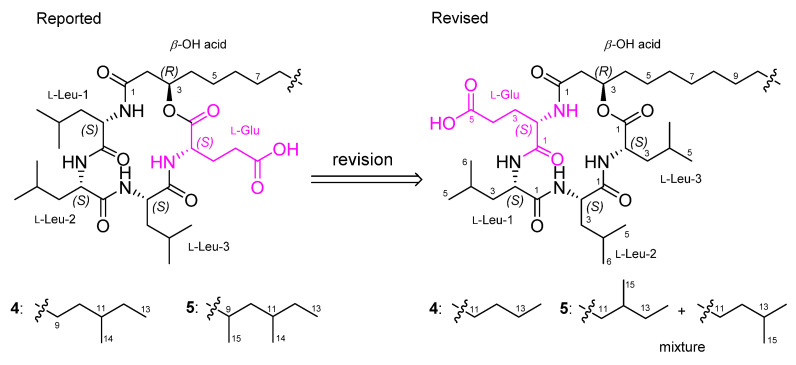
Reported and revised structures of bacilotetrins A (**4**) and B (**5**).

As the spectroscopic data, including NMR and MS, of **1**–**3** were very similar to previously reported bacilotetrins A (**4**) and B (**5**), we carefully compared and checked the NMR data of these compounds to discover that the planar structures of **4** and **5** were incorrectly determined. In the original paper for **4**, the NMR signals for the carbonyl carbon (*δ*_C_ 173.0) and *α*-position (*δ*_C_ 51.6 and *δ*_H_ 4.58) of Leu-3 were misassigned to those of Glu (Table S4). These misassignments led to a wrong determination of the sequence of amino acids in **4**. In addition, the methine signals (*δ*_C_ 30.6 and *δ*_H_ 1.50) for the anteiso-type *β*-OH acid in the original NMR data of **4** were not true signals. The methyl signals (*δ*_C_ 14.6 and *δ*_H_ 0.89) of the *β*-OH acid in **4** were in good agreement with those (*δ*_C_ 14.5 and *δ*_H_ 0.89) of **1** and the literature values for the linear-type *β*-OH acid [19]. Thus, we revise the planar structure of **4** to have a linear-type *β*-OH acid instead of the anteiso-type *β*-OH acid, and to be cyclo-(*R*-*β*-OH acid-L-Glu-L-Leu-L-Leu-L-Leu) instead of cyclo-(*R*-*β*-OH acid-L-Leu-L-Leu-L-Leu-L-Glu) (Figure 5). Compound **5** was reported to have a 3-hydroxy-9,11-dimethyltridecanoic acid (HDTA, C_15_H_30_O_3_) as a *β*-OH acid. However, by the detailed analysis of 2D NMR data, we found that the HDTA unit in **5** is a mixture of a 3-hydroxy-12-methyltetradecanoic acid and a 3-hydroxy-13-methyltetradecanoic acid, which have the same molecular weight and formula to HDTA, as shown in Figure 5. This fact was also supported by the chemical shifts of the methyl signals (*δ*_C_ 19.8/*δ*_H_ 0.86, *δ*_C_ 11.9/*δ*_H_ 0.87, and *δ*_C_ 23.1/*δ*_H_ 0.87) of the *β*-OH acid in **5**, which were well matched with the literature values for the anteiso- and iso-type *β*-OH acids [19]. Therefore, the planar structures of **4** and **5** should be revised to have the same planar core structure as baciloterins C–E (**1**–**3**). However, **4** and **5** consist of only L-amino acids, whereas **1**–**3** consist of L- and D-amino acids, and these compounds had slightly different optical rotation values (**1**: [*α*${]}_{D}^{25}$ −50 (*c* 0.1, MeOH); **4**: [*α*${]}_{D}^{25}$ −22.1 (*c* 0.05, MeOH) and **2**: [*α*${]}_{D}^{25}$ −70 (*c* 0.1, MeOH); **3**: [*α*${]}_{D}^{25}$ −63 (*c* 0.1, MeOH); **5**: [*α*${]}_{D}^{25}$ −18.6 (*c* 0.05, MeOH)). Therefore, these facts revealed that **1** is a new diastereomeric isomer of **4**, and **2** and **3** are new diastereomers of **5** (Figure 5).

The structures of **1**–**3** have a similar structural composition to surfactins. Surfactins are cyclic lipopeptides consisting of seven amino acids (L-Glu-L-Leu-D-Leu-L-Val-L-Asp-D-Leu-L-Leu) and a *β*-OH fatty acid having 13 to 15 carbon atoms [22]. Likewise, **1**–**3** are also cyclic lipopeptides consisting of four amino acids (L-Glu-L-Leu-D-Leu-L-Leu) and a *β*-OH acid having 14 or 15 carbon atoms in a similar manner. These structural similarities suggest that **1**–**3** might be biosynthesized by a similar biosynthetic pathway, a non-ribosomal peptide synthetase (NRPS), to surfactins. The cyclic lipopeptide surfactins are synthesized by a complex of three surfactin synthetase subunits SrfA-A, SrfA-B, and SrfA-C [23]. These subunits consist of either three modules (SrfA-A and SrfA-B) or one module (SrfA-C) and each module contributes to the addition of one amino acid [24]. In the case of **1**–**3**, it is predicted that one SrfA-B module is omitted and other modules are related to produce the structures (Figure S37).

### 2.2. Inhibitory Activity of Isolated Compounds against Mycoplasma hyorhinis

The anti-mycoplasma activity of **1**–**3** was assessed by broth dilution assay (Table 2). Compounds **1**–**3** exhibited anti-mycoplasma activity, with an MIC value of 31 μg/mL. These results revealed that the type of branch of *β*-OH fatty acids does not affect their inhibitory activity against *M. hyorhinis*, and the cyclic lipodepsipeptide core plays a more important role.

## 3. Materials and Methods

### 3.1. General Experimental Procedures and Reagents

UV spectra were recorded with a Shimadzu UV-1650PC spectrophotometer (Shimadzu Corporation, Kyoto, Japan). IR spectra were obtained on a JASCO FT/IR-4100 spectrophotometer (JASCO Corporation, Tokyo, Japan). Optical rotations were measured with a Rudolph analytical Autopol III S2 polarimeter (Rudolph Research Analytical, Hackettstown, NJ, USA). NMR spectra were acquired with a Bruker AVANCE III 600 spectrometer (Bruker BioSpin GmbH, Rheinstetten, Germany) with a 3 mm probe operating at 600 MHz (^1^H) and 150 MHz (^13^C). Chemical shifts were expressed in ppm with reference to the solvent peaks (*δ*_H_ 3.31 and *δ*_C_ 49.15 ppm for CD_3_OH). LR-EIMS and Marfey’s analysis data were acquired using an Agilent 6100 single quadrupole mass spectrometer (Agilent Technologies, Santa Clara, CA, USA). HR-ESIMS data were obtained with a Waters SYNPT G2 Q-TOF mass spectrometer (Waters Corporation, Milford, CT, USA) at Korea Basic Science Institute (KBSI) in Cheongju, Republic of Korea. HPLC was performed using a PrimeLine binary pump (Analytical Scientific Instruments, Inc., El Sobrante, CA, USA) with Shodex RI-101 refractive index detector (Shoko Scientific Co. Ltd., Yokohama, Japan) and S3210 variable UV detector (Schambeck SFD GmbH, Bad Honnef, Germany), along with Thermo Fisher Scientific UltiMate 3000 HPLC system (Thermo Scientific, Bremen, Germany). Columns for HPLC were YMC-ODS-A (250 mm × 10 mm, 5 μm; and 250 mm × 10 mm, 5 μm) and YMC-Triart C_18_ (250 mm × 10 mm, 5 μm; and 250 mm × 10 mm, 5 μm). C_18_-reversed-phase silica gel (YMC-Gel ODS-A, 12 nm, S-75 μm) was used for open column chromatography. Organic solvents were purchased as HPLC grade from Duksan (Ansan, Republic of Korea) and Samchun (Pyeongtaek, Republic of Korea). Pure and ultrapure waters were obtained from the Milipore Mili-Q Direct 8 system (Milipore S.A.S. Molsheim, France).

### 3.2. Micro-Organism and Fermentation

The bacterial strain *Bacillus subtilis* 109GGC020 (Genbank accession number JQ927413) was isolated from a marine sponge sample collected from the Gageo reef, Republic of Korea in 2010. The seed culture and production cultures were carried out in Bennett (BN)’s broth [9] (1% glucose, 0.2% tryptone, 0.1% yeast extract, 0.1% beef extract, 0.5% glycerol, 1.85% artificial sea salt, pH 7 before sterilization). The seed culture was performed in a 250 mL Erlenmeyer flask containing 100 mL BN broth at 28 °C, 120 rpm for 3 days. The seed culture was inoculated into a 100 L fermenter containing 70 L of the broth medium under the aseptic condition. The fermenter was operated at 28 °C, 55 rpm, and airflow rate of 20 L/min (LPM) for 7 days. The culture broth was separated by high-speed centrifugation (60,000 rpm) into cell mass and broth. The broth part was extracted with an equal volume of ethyl acetate (EtOAc, 70 L) twice.

### 3.3. Extraction and Isolation of Compounds ***1***–***3***

The EtOAc extract was concentrated in vacuo, and 28.4 g of a crude extract was obtained. A portion of the crude extract (9.7 g) was subjected to reversed-phase vacuum column chromatography (YMC Gel ODS-A, 12 nm, S 75 μm) with a stepwise gradient solvent system of 20, 40, 60, 80, and 100% MeOH in H_2_O. The 100% MeOH fraction, showing characteristic two exchangeable proton signals in 7–8 ppm of bacilotetrins, was selected for further purification.

The 100% MeOH fraction (2.3 g) was applied to ODS vacuum column chromatography, followed by a stepwise gradient elution with 80, 90, and 100% MeOH in H_2_O. In addition, each fraction was divided into three subfractions. Subfraction-3 of the 90% MeOH fraction (1.5 g) was purified by reversed-phase HPLC (YMC ODS-A, 250 × 10 mm, 5 μm, 86% MeOH in H_2_O, 2.0 mL/min, RI, runtime 60 min) to obtain **1** (19.1 mg, *t*_R_ 37 min). Subfraction-1 of the 100% MeOH fraction (200 mg) was purified by HPLC (YMC Triart C_18_, 250 × 10 mm, 5 μm, 90% MeOH in H_2_O, 2.0 mL/min, RI, runtime 55 min) to yield a subfraction containing **2** and **3**. The subfraction was again subjected to further purification by HPLC (YMC Triart C_18_, 250 × 4.6 mm, 5 μm, 70% MeCN in H_2_O + 0.01% TFA, 0.7 mL/min, UV: 224 nm, runtime 60 min) to obtain **2** (3.7 mg, *t*_R_ 49 min) and **3** (2.7 mg, *t*_R_ 51 min).

Bacilotetrin C (**1**): Amorphous solid; [*α*${]}_{D}^{25}$ −50 (*c* 0.1, MeOH); IR (MeOH) *γ*_max_ 3297, 2925, 1643, 1052 cm^−1^; ^1^H and ^13^C NMR data, see Table 1; HR-ESIMS *m*/*z* [M + Na]^+^ 717.4775 (calculated for C_37_H_66_N_4_O_8_Na, 717.4778).

Bacilotetrin D (**2**): Amorphous solid; [*α*${]}_{D}^{25}$ −70 (*c* 0.1, MeOH); IR (MeOH) *γ*_max_ 3300, 2957, 1653, 1057 cm^−1^; ^1^H and ^13^C NMR data, see Table 1; HR-ESIMS *m*/*z* [M + Na]^+^ 731.4934 (calculated for C_38_H_68_N_4_O_8_Na, 731.4935).

Bacilotetrin E (**3**): Amorphous solid; [*α*${]}_{D}^{25}$ −63 (*c* 0.1, MeOH); IR (MeOH) *γ*_max_ 3297, 2961, 1650, 1057 cm^−1^; ^1^H and ^13^C NMR data, see Table 1; HR-ESIMS *m*/*z* [M + Na]^+^ 731.4937 (calculated for C_38_H_68_N_4_O_8_Na, 731.4935).

### 3.4. Total Hydrolysis and Marfey’s Analysis

Compound **1** (0.4 mg) was treated with 6N HCl (300 μL) for 12 h at 110 °C. The completion of reaction was confirmed by LR-LCMS analysis. The reaction mixture was cooled down to room temperature, and partitioned with water and hexane (Hex). The aqueous layer was evaporated and treated with 0.1% 1-fluoro-2,4-dinitro-phenyl-5-L-leucinamide (L-FDLA, 600 μL) in acetone and 1M NaHCO_3_ (120 μL). The mixture was stirred at 40 °C for 1 h. After cooling to room temperature, the solution was neutralized with 1N HCl (120 μL) and diluted with MeCN (420 μL). Likewise, standard L- and D-amino acids were derivatized with L-FDLA, following the above-mentioned method. Marfey’s derivative of **1** was analyzed by LR-LCMS (YMC ODS-A, 250 × 4.6 mm, 5 μm, 0.5 mL/min, UV: 340 nm) using a gradient MeCN–H_2_O (+0.02% TFA) solvent system (40% MeCN for 5 min, 40–80% MeCN over 20 min, and 80% MeCN for 5 min), and the retention time was compared with authentic standard derivatives (Figure S25 and Table S1). As a result, the composition of amino acid from **1** was confirmed as L-Glu (16.9 min), L-Leu (23.6 min), and D-Leu (29.0 min). Standard amino acid derivatives with L-FDLA: L-Glu (16.9 min), D-Glu (17.8 min), L-Leu (23.6 min), and D-Leu (28.9 min).

### 3.5. Partial Hydrolysis and Marfey’s Analysis

Compound **1** (2.0 mg) was treated with 1 mL of 4N HCl:AcOH (1:1) at 100 °C for 2 h. The reaction was monitored by LR-LCMS. The hydrolysate was concentrated under a N_2_ stream, and partitioned with H_2_O and hexane. The aqueous layer was concentrated in vacuo and purified by LR-LCMS (YMC ODS-A, 250 × 4.6 mm, 5 μm, 0.5 mL/min, UV: 224 nm) using a gradient MeCN–H_2_O (+0.02% TFA) solvent system (20% MeCN for 10 min, 20–100% MeCN over 40 min, and 100% MeCN for 10 min) to obtain three fragments (P1: Glu–Leu, *t*_R_ 7.6 min, *m*/*z* 261 [M + H]^+^; P2: Leu–Leu, *t*_R_ 23.0 min, *m*/*z* 245 [M + H]^+^; P3: *β*-OH-acid–Leu, *t*_R_ 28.6 min, *m*/*z* 358 [M + H]^+^) (Figure S26). Two fragments, P1 (Glu–Leu) and P3 (*β*-OH-acid–Leu), were subjected to total hydrolysis and derivatization with L-FDLA, which were analyzed by LR-LCMS, as previously described (Figure S27 and Table S2). Both leucines from fragments P1 and P3 were identified as L-form (hydrolysate of P1-L-FDLA: *t*_R_ 23.7 min, hydrolysate of P3-L-FDLA: *t*_R_ 23.7 min). The other fragment P2 (Leu–Leu) was reacted with L-FDLA, which was analyzed by comparing the retention time with L-FDLA derivatives of standard reagents (L-Leu-D-Leu and D-Leu-L-Leu, Figure S28) (Figure S29 and Table S3). The Leu–Leu of P2 was determined as a mixture of L-Leu-D-Leu and D-Leu-L-Leu (P2-L-FDLA: *t*_R_ 26.1 min (major) and *t*_R_ 31.6 min (minor), *m*/*z* 589 [M + H]^+^, L-Leu-D-Leu-L-FDLA: *t*_R_ 26.1 min, *m*/*z* 589 [M + H]^+^, D-Leu-L-Leu-L-FDLA: *t*_R_ 31.6 min, *m*/*z* 589 [M + H]^+^).

### 3.6. Methanolysis of ***1***

Compound **1** (2.4 mg) was dissolved in 1.2 mL of 3M methanolic HCl and refluxed for 2 h. The completion of reaction was confirmed by LR-LCMS analysis. The mixture was concentrated under a N_2_ gas stream and partitioned with Hex and water. The Hex layer was dried and 1.0 mg of a crude fatty acid ester **1a** was obtained (Figure S34).

### 3.7. Preparation of the (S)- and (R)-MTPA Esters (***1b*** and ***1c***)

Crude fatty acid ester **1a** was divided equally into two portions and dried under a N_2_ gas stream. A few crystals of 4-dimethylaminopyridine (DMAP) and anhydrous pyridine (80 μL) were added to each vial and stirred at room temperature for 5 min. Then, 5 μL of *R*-(−) or *S*-(+)-*α*-methoxy-*α*-(trifluoromethyl)phenylacetyl chloride (MTPA-Cl) was added, respectively. The mixtures were stirred at room temperature for 16 h. The reaction mixtures were concentrated under a N_2_ gas steam at 40 °C. Each mixture was dissolved in methylene chloride (MC) and washed with 1N HCl solution, saturated NaHCO_3_ solution and brine. The MC layer was dried over anhydrous MgSO_4_ and evaporated in vacuo. Each residue was purified by reversed-phase HPLC (YMC-Triart C_18_, 250 × 4.6 mm, 5 μm, 1.0 mL/min, UV: 210 and 254 nm) using a gradient MeCN–H_2_O solvent system (40% MeCN for 5 min, 40–100% MeCN over 30 min, and 100% MeCN for 10 min) to give (*S*)-MTPA ester **1b** (0.2 mg, *t*_R_ 39.4 min) and (*R*)-MTPA ester **1c** (0.3 mg, *t*_R_ 39.6 min). The ^1^H chemical shifts around the stereogenic center of MTPA esters were confirmed by the analysis of ^1^H and COSY spectrum (Figures S30−S33).

*S*-MTPA ester of **1a** (**1b**): ^1^H NMR (600 MHz, CDCl_3_) *δ* 5.45 (m, H-3), 3.57 (s, OCH_3_), 2.62 (dd, *J* = 15.9, 8.0 Hz, H-2a), 2.56 (dd, *J* = 15.9, 5.0 Hz, H-2b), 1.72 (m, H-4a), 1.64 (m, H-4b); EIMS *m*/*z* [M + Na]^+^ 497.3.

*R*-MTPA ester of **1a** (**1c**): ^1^H NMR (600 MHz, CDCl_3_) *δ* 5.45 (m, H-3), 3.64 (s, OCH_3_), 2.67 (dd, *J* = 15.9, 8.3 Hz, H-2a), 2.60 (dd, *J* = 15.9, 4.6 Hz, H-2b), 1.63 (m, H-4a), 1.58 (m, H-4b); EIMS *m*/*z* [M + Na]^+^ 497.2.

### 3.8. Measurement of Anti-Mycoplasma Activity

Anti-mycoplasma activity against *Mycoplasma hyorhinis* of **1**–**3** was evaluated by broth dilution assay. In brief, the test strain, *Mycoplasma hyorhinis* ATCC 17981, was cultured in PPLO broth medium [25] at 37 °C under a humidified atmosphere of 5% CO_2_. Stock solutions of **1**–**3** were dissolved in DMSO and diluted with PPLO broth medium to give serial twofold dilutions in the range of 500 to 1 μg/mL. The final DMSO concentration was maintained at 5% by adding DMSO to the PPLO broth. Culture broth (100 μL) containing approximately 2 × 10^4^ CFU/mL of activated strain was added to each well of a 96-well plate. The plates were incubated for 7 days at 37 °C under a humidified atmosphere of 5% CO_2_. The color of the broth changes to yellow as bacteria grow. The minimum inhibitory concentration (MIC) values were determined as the lowest concentration of the test compound that inhibited bacterial growth. BioMycoX^®^ (CellSafe Co., Yongin, Republic of Korea) was used for a positive control.

## 4. Conclusions

Three new cyclic lipodepsipeptides, bacilotetrins C–E, consisting of four amino acids and a *β*-hydroxy fatty acid were isolated from the culture broth of *Bacillus subtilis* 109GGC020. Their spectroscopic data were very similar to those of previously reported for bacilotetrins A (**4**) and B (**5**). By the detailed and careful analysis of NMR data, we found that the planar structures of **4** and **5** must be reassigned and our comprehensive spectroscopic data analysis led to revision of their structures. In the revised structures of **4** and **5**, the positions of Glu and the branch types of the *β*-hydroxy fatty acids are correctly determined.

The absolute configurations of the amino acids and *β*-hydroxy fatty acid in **1**–**3** were established by chemical derivatization, including Marfey’s and Mosher’s methods. The major difference between **1**–**3** and **4**–**5** lay in the fact that **1**–**3** consist of L- and D-amino acids, whereas **4**–**5** have only L-amino acids. In addition, as previously mentioned, **1**–**3** were expected to be synthesized through the similar biosynthetic pathway to surfactins, which are well known for their various biological activities, such as antifungal, antibacterial, anticancer, and anti-mycoplasma activities [22]. Compounds **1**–**3** also showed antimicrobial activity against *M. hyorhinis*, which is known to cause diseases, such as polyserositis, arthritis, conjunctivitis, and otitis in pigs, with an MIC value of 31 μg/mL, twofold stronger than that of the positive control, BioMycoX^®^. The only difference between **1**–**3** was the type of branch in the *β*-OH acid. Therefore, on the basis of the result, it is supposed that the cyclic peptide core plays an important role in anti-mycoplasma activity, but the type of branch in the *β*-OH acid is not critical for activity. Further studies are needed to clarify the underlying mechanism of the activity for the development of antibiotics.

## Figures and Tables

**Table 1:** ^1^H and ^13^C NMR data for compounds **1**–**3** (600 MHz, CD_3_OH).

Position	1		2		3	
	*δ*_C_, Type	*δ*_H_, m (*J* in Hz)	*δ*_C_, Type	*δ*_H_, m (*J* in Hz)	*δ*_C_, Type	*δ*_H_, m (*J* in Hz)
Glu						
1	175.9, C		175.9, C		175.9, C	
2	55.6, CH	4.11, td (7.3, 4.0)	55.5, CH	4.11, m	55.5, CH	4.11, m
3	27.3, CH_2_	1.95, q (7.5)	27.3, CH_2_	1.95, q (7.5)	27.3, CH_2_	1.94, q (7.4)
4	31.1, CH_2_	2.42, m	31.1, CH_2_	2.43, m	31.1, CH_2_	2.42, m
5	176.3, C		176.3, C		176.3, C	
NH		8.39, d (4.7)		8.42, d (4.5)		8.41, d (4.5)
Leu-1						
1	173.9, C		173.9, C		173.9, C	
2	55.0, CH	3.74, m	55.0, CH	3.75, m	55.0, CH	3.74, m
3	38.5, CH_2_	2.01, ddd (14.6, 11.0, 3.9) 1.80, o.l *^a^*	38.5, CH_2_	2.01, m 1.80, o.l *^a^*	38.5, CH_2_	2.01, m 1.80, o.l *^a^*
4	26.3, CH	1.61, m	26.3, CH	1.60, o.l *^a^*	26.3, CH	1.60, o.l *^a^*
5	21.3, CH_3_	0.93, d (5.8)	21.3, CH_3_	0.93, d (5.4)	21.3, CH_3_	0.94, d (5.3)
6	24.0, CH_3_	0.95, d (5.8)	24.0, CH_3_	0.95, d (5.4)	24.0, CH_3_	0.95, d (5.3)
NH		9.10, d (6.7)		9.13, d (6.6)		9.12, d (6.7)
Leu-2						
1	174.5, C		174.5, C		174.5, C	
2	53.4, CH	4.43, m	53.3, CH	4.42, m	53.4, CH	4.42, m
3	40.4, CH_2_	1.81, o.l *^a^*	40.4, CH_2_	1.76, o.l *^a^*	40.4, CH_2_	1.78, o.l *^a^*
4	26.2, CH	1.70, m	26.2, CH	1.71, o.l *^a^*	26.2, CH	1.71, o.l *^a^*
5	21.2, CH_3_	0.90, d (6.4)	21.2, CH_3_	0.90, d (6.4)	21.2, CH_3_	0.91, d (6.4)
6	23.9, CH_3_	0.96, d (6.4)	23.9, CH_3_	0.96, d (6.4)	23.9, CH_3_	0.98, d (6.4)
NH		7.74, d (8.6)		7.75, d (8.5)		7.74, d (8.8)
Leu-3						
1	172.9, C		173.0, C		173.0, C	
2	51.6, CH	4.57, m	51.6, CH	4.57, m	51.6, CH	4.57, m
3	40.6, CH_2_	1.80, m 1.69, m	40.6, CH_2_	1.81, m 1.69, m	40.6, CH_2_	1.80, m 1.70, m
4	25.8, CH	1.65, o.l *^a^*	25.7, CH	1.65, o.l *^a^*	25.7, CH	1.66, m
5	21.8, CH_3_	0.89, o.l *^a^*	21.7, CH_3_	0.89, d (6.4)	21.7, CH_3_	0.90, d (6.5)
6	23.8, CH_3_	0.92, d (6.5)	23.7, CH_3_	0.92, d (6.4)	23.7, CH_3_	0.93, d (6.5)
NH		7.76, d (9.5)		7.77, d (9.5)		7.77, d (9.4)
*β*-OH acid						
1	173.3, C		173.3, C		173.0, C	
2	41.5, CH_2_	2.72, dd (13.8, 4.6) 2.29, dd (13.8, 8.1)	41.5, CH_2_	2.72, dd (13.8, 4.6) 2.29, dd (13.8, 8.1)	41.5, CH_2_	2.72, dd (13.8, 4.7) 2.29, dd (13.8, 8.1)
3	73.8, CH	5.16, tt (7.8, 5.3)	73.8, CH	5.15, m	73.8, CH	5.15, m
4	35.5, CH_2_	1.80, o.l *^a^* 1.57, o.l *^a^*	35.4, CH_2_	1.81, o.l *^a^* 1.56, o.l *^a^*	35.4, CH_2_	1.81, o.l *^a^* 1.62, o.l *^a^*
5	26.4– 30.8, CH_2_	1.29, o.l *^a^*	28.2– 30.6, CH_2_	1.29, o.l *^a^*	26.3, CH_2_	1.28, o.l *^a^*
6					28.6– 31.0, CH_2_	1.28, o.l *^a^*
7						
8						
9						
10						
11			30.7, CH_2_	1.34, o.l *^a^* 1.13, o.l *^a^*		
12	33.1, CH_2_	1.27, o.l *^a^*	35.7, CH	1.29, o.l *^a^*	40.3, CH_2_	1.16, m
13	23.8, CH_2_	1.30, o.l *^a^*	37.8, CH_2_	1.29, o.l *^a^* 1.09, o.l *^a^*	29.2, CH	1.51, m
14	14.5, CH_3_	0.89, o.l *^a^*	11.8, CH_3_	0.87, o.l *^a^*	23.1, CH_3_	0.87, d (6.4)
15			19.7, CH_3_	0.85, d (4.8)	23.1, CH_3_	0.87, d (6.4)

*^a^* Signals were overlapped with other signals.

**Table 2:** MIC values of bacilotetrins C–E (**1**–**3**).

	Compounds			
	1	2	3	BioMycoX^® 1^
MIC (μg/mL)	31	31	31	62

^1^ Positive control.

## Data Availability

The Data presented in the article are available in the supplementary materials.

## Supplementary Materials

The following are available online at https://www.mdpi.com/article/10.3390/md19100528/s1, Figures S1–S8: ^1^H, ^13^C, HSQC, COSY, TOCSY, HMBC, NOESY and HR-ESIMS data of **1**, Figures S9–S16: ^1^H, ^13^C, HSQC, COSY, TOCSY, HMBC, NOESY and HR-ESIMS data of **2**, Figures S17–S24: ^1^H, ^13^C, HSQC, COSY, TOCSY, HMBC, NOESY and HR-ESIMS data of **3**, Figure S25 and Table S1: HPLC analysis of amino acids in **1** through the total hydrolysis and retention times of L-FDLA derivatives of hydrolysate of **1**, Figure S26: HPLC chromatogram of partial hydrolysis of **1**, Figure S27 and Table S2: HPLC analysis of L-FDLA derivatives of P1 and P3 hydrolysates and retention times of L-FDLA derivatives of hydrolysate of P1 and P3 hydrolysates, Figure S28: Structures of standard dipeptides, Figure S29 and Table S3: HPLC analysis of L-FDLA derivatives of P2 and retention times of L-FDLA derivatives of P2, Figures S30–S33: ^1^H and COSY spectrum of MTPA esters (**1b** and **1c**), Figures S34–36: LR-EIMS spectrum of crude **1a** and MTPA esters (**1b** and **1c**), Figure S37: Plausible biosynthetic pathway of compounds **1**–**3** and biosynthetic pathway of surfactin, Table S4: ^1^H and ^13^C NMR data for reported and revised bacilotetrin A (**4**).
