# Supplementary material for: Anti-Mycoplasma Activity of Bacilotetrins C–E, Cyclic Lipodepsipeptides from the Marine-Derived Bacillus subtilis and Structure Revision of Bacilotetrins A and B
Source: Mar Drugs. 2021 Sep 22;19(10):528. doi: 10.3390/md19100528 (PMC8537332; doi:10.3390/md19100528)
Supplement: Supplementary file 1 [file marinedrugs-19-00528-s001.zip › marinedrugs-1376842-supplementary.pdf]

Supporting Information

Anti-Mycoplasma Activity of Bacilotetrins C–E, Cyclic Lipodepsipeptides from the Marine-Derived *Bacillus subtilis* and Structure Revision of Bacilotetrins A and B

Hwa-Sun Lee and Hee Jae Shin *

Marine Natural Products Chemistry Laboratory, Korea Institute of Ocean Science & Technology, 385 Haeyang-ro, Yeongdo-gu, Busan 49111, Korea; hwasunlee@kiost.ac.kr
* Correspondence: shinhj@kiost.ac.kr; Tel.: +82-51-664-3341

Contents **Page**

Figure S1.	^{1}H NMR spectrum of compound **1** (600 MHz, CD_3OH).	S3
Figure S2.	^{13}C NMR spectrum of compound **1**(150 MHz, CD_3OH).	S3
Figure S3.	HSQC spectrum of compound **1** in CD_3OH.	S4
Figure S4.	COSY spectrum of compound **1** in CD_3OH.	S4
Figure S5.	TOCSY spectrum of compound **1** in CD_3OH.	S5
Figure S6.	HMBC spectrum of compound **1** in CD_3OH.	S5
Figure S7.	NOESY spectrum of compound **1** in CD_3OH.	S6
Figure S8.	HR-ESIMS spectrum of compound **1**.	S7
Figure S9.	^{1}H NMR spectrum of compound **2** (600 MHz, CD_3OH).	S8
Figure S10.	^{13}C NMR spectrum of compound **2** (150 MHz, CD_3OH).	S8
Figure S11.	HSQC spectrum of compound **2** in CD_3OH.	S9
Figure S12.	COSY spectrum of compound **2** in CD_3OH.	S9
Figure S13.	TOCSY spectrum of compound **2** in CD_3OH.	S10
Figure S14.	HMBC spectrum of compound **2** in CD_3OH.	S10
Figure S15.	ROESY spectrum of compound **2** in CD_3OH.	S11
Figure S16.	HR-ESIMS spectrum of compound **2**.	S12

Contents		Page
Figure S17.	^1H NMR spectrum of compound **3** (600 MHz, CD_3OH).	S13
Figure S18.	^{13}C NMR spectrum of compound **3** (150 MHz, CD_3OH).	S13
Figure S19.	HSQC spectrum of compound **3** in CD_3OH.	S14
Figure S20.	COSY spectrum of compound **3** in CD_3OH.	S14
Figure S21.	TOCSY spectrum of compound **3** in CD_3OH.	S15
Figure S22.	HMBC spectrum of compound **3** in CD_3OH.	S15
Figure S23.	ROESY spectrum of compound **3** in CD_3OH.	S16
Figure S24.	HR-ESIMS spectrum of compound **3**.	S17
Figure S25.	HPLC analysis of amino acids in **1** through the total hydrolysis.	S18
Table S1.	Retention times (t_R, min) of L-FDLA derivatives of the hydrolysate of **1**	S18
Figure S26.	HPLC-VWD-MS chromatogram of the partial hydrolysate of **1**.	S19
Figure S27.	HPLC analysis of L-FDLA derivatives of P1 and P3 hydrolysates.	S19
Table S2.	Retention times (t_R, min) of L-FDLA derivatives of P1 and P3 hydrolysates	S19
Figure S28.	Structures of standard dipeptides.	S20
Figure S29.	HPLC analysis of L-FDLA derivatives of P2.	S20
Table S3.	Retention times (t_R, min) of L-FDLA derivatives of P2	S20
Figure S30.	^1H NMR spectrum of the (*S*)-MTPA ester (**1b**) (600 MHz, $CDCl_3$).	S21
Figure S31.	COSY spectrum of the (*S*)-MTPA ester (**1b**) in $CDCl_3$.	S21
Figure S32.	^1H NMR spectrum of the (*R*)-MTPA ester (**1c**) (600 MHz, $CDCl_3$).	S22
Figure S33.	COSY spectrum of the (*R*)-MTPA ester (**1c**) in $CDCl_3$.	S22
Figure S34.	LR-EIMS spectrum of **1a**.	S23
Figure S35.	LR-EIMS spectrum of the (*S*)-MTPA ester (**1b**).	S23
Figure S36.	LR-EIMS spectrum of the (*R*)-MTPA ester (**1c**).	S23
Figure S37.	Plausible biosynthetic pathway of compounds **1**–**3**.	S24
Table S4.	^1H and ^{13}C NMR data for data for reported and revised bacilotetrin A (**4**) (500 MHz, CD_3OD)	S25

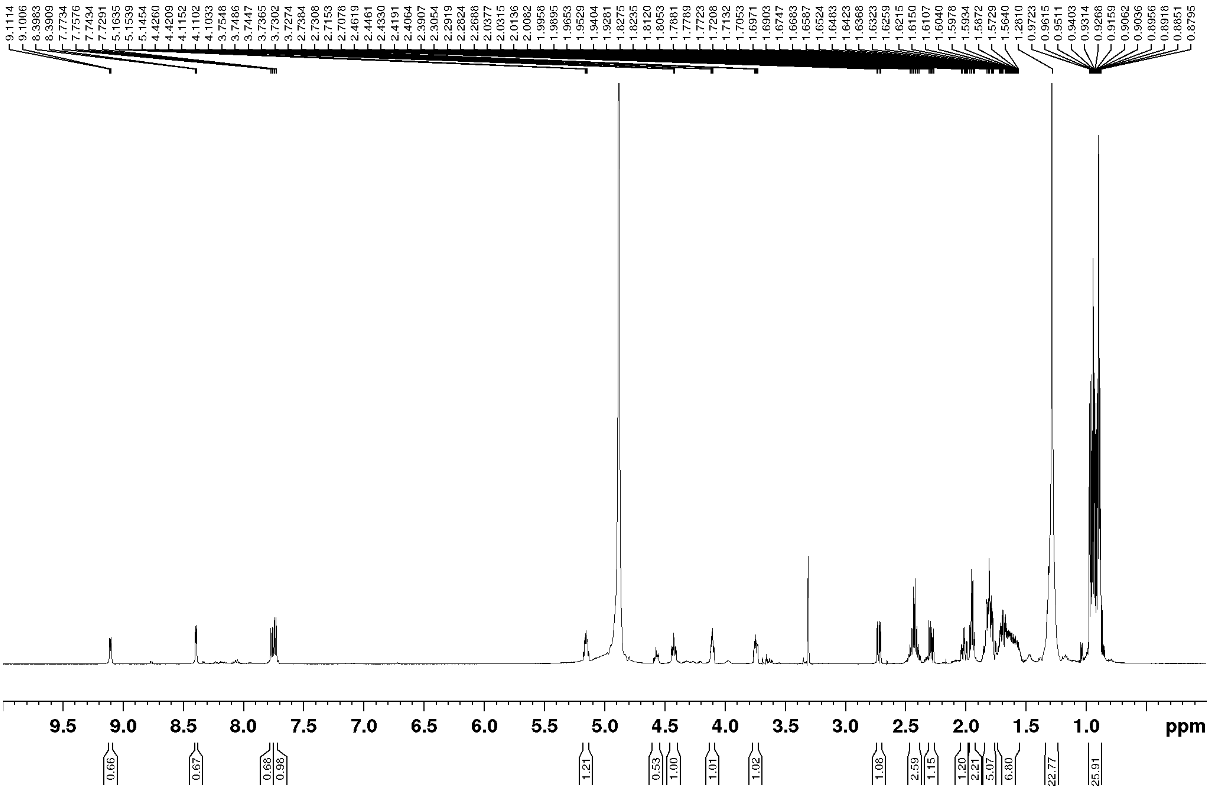

Figure S1. ^{1}H NMR spectrum of compound **1** (600 MHz, CD_3OH).

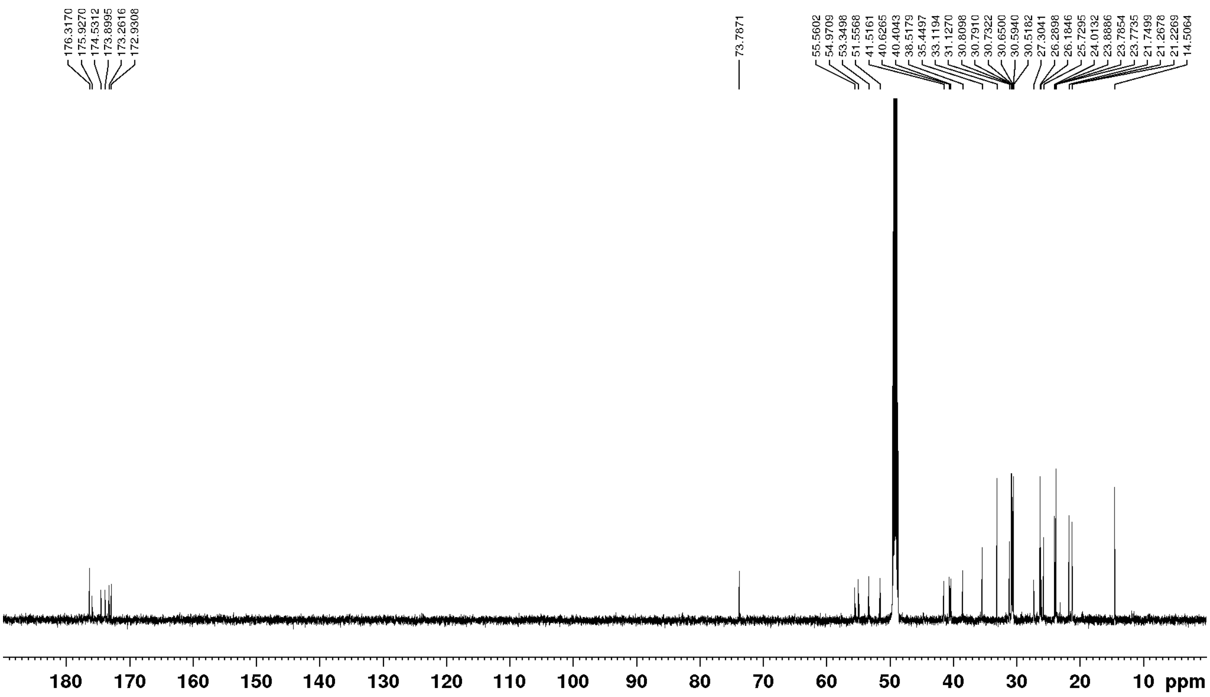

Figure S2. ^{13}C NMR spectrum of compound **1** (150 MHz, CD_3OH).

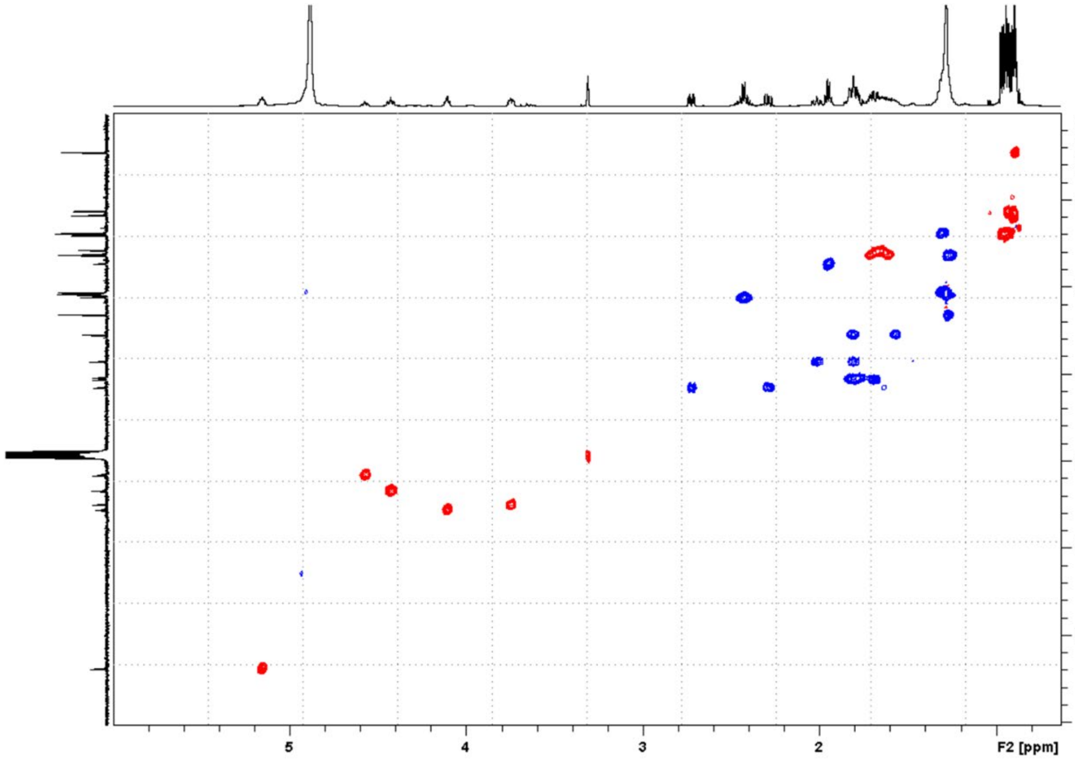

Figure S3. HSQC spectrum of compound **1** in CD_3OH.

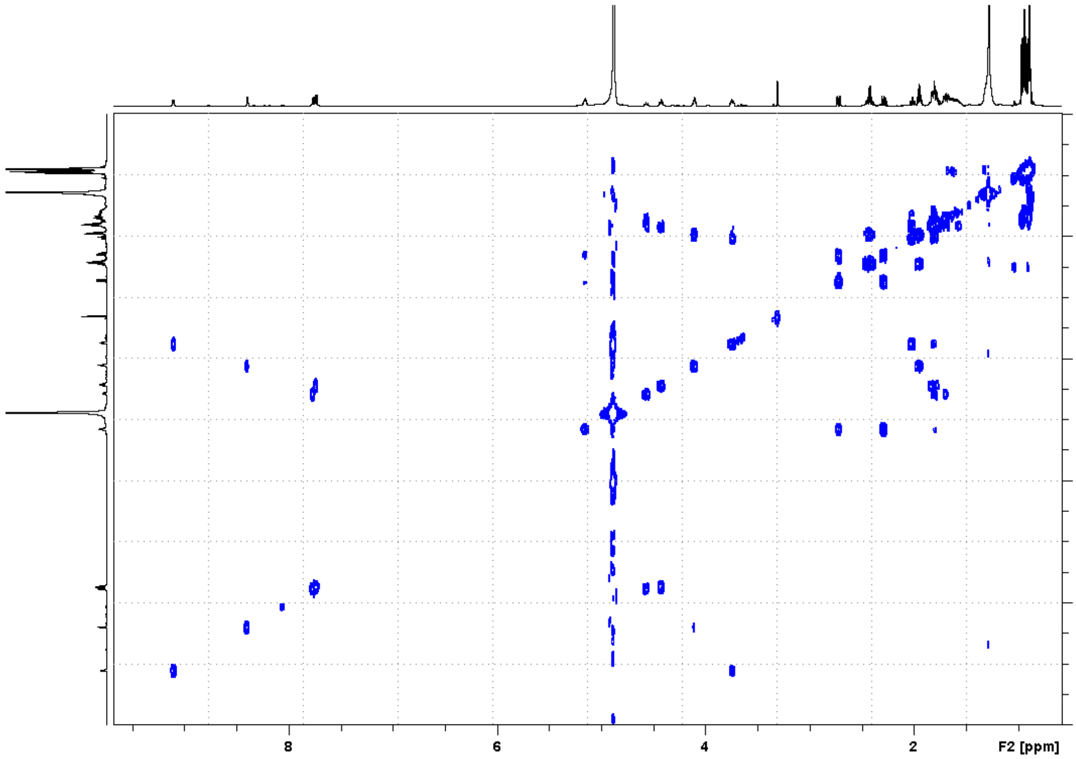

Figure S4. COSY spectrum of compound **1** in CD_3OH.

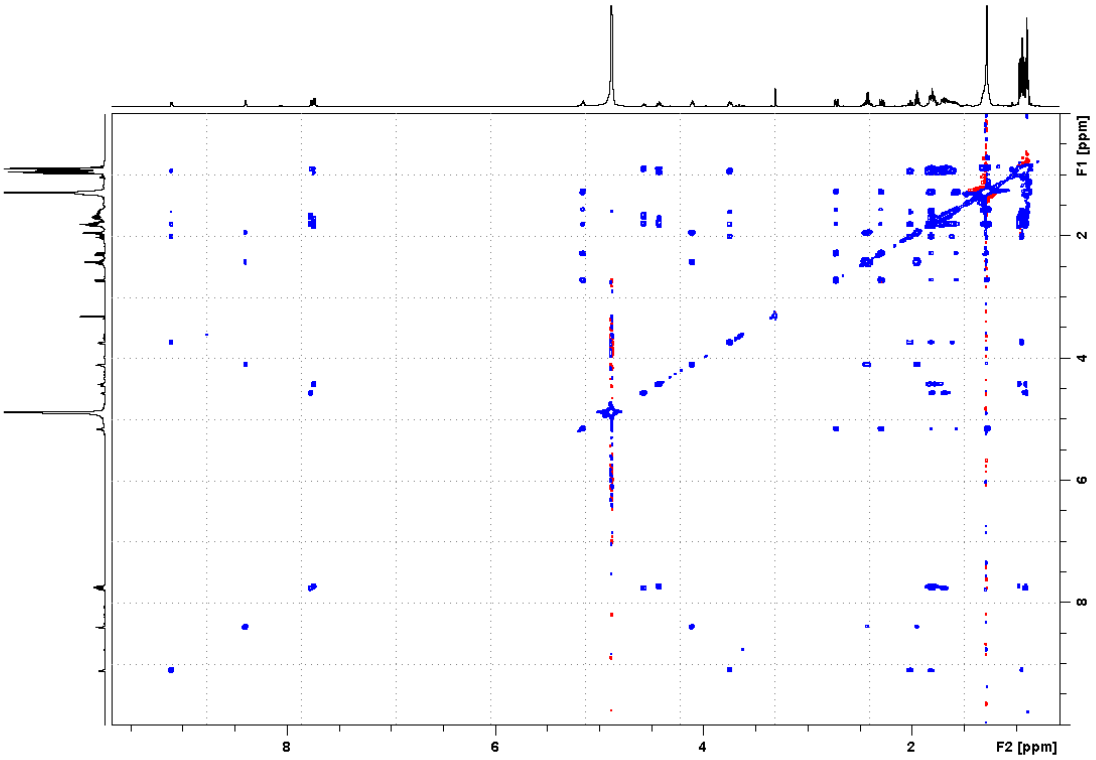

Figure S5. TOCSY spectrum of compound **1** in CD_3OH.

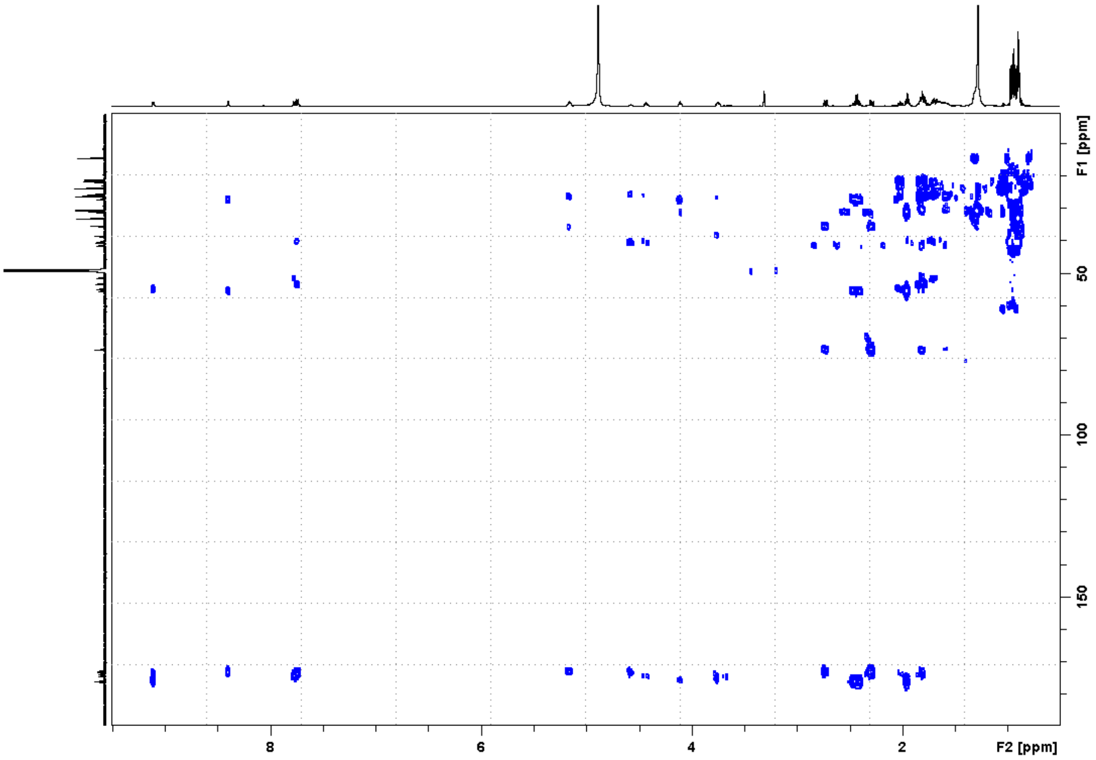

Figure S6. HMBC spectrum of compound **1** in CD_3OH.

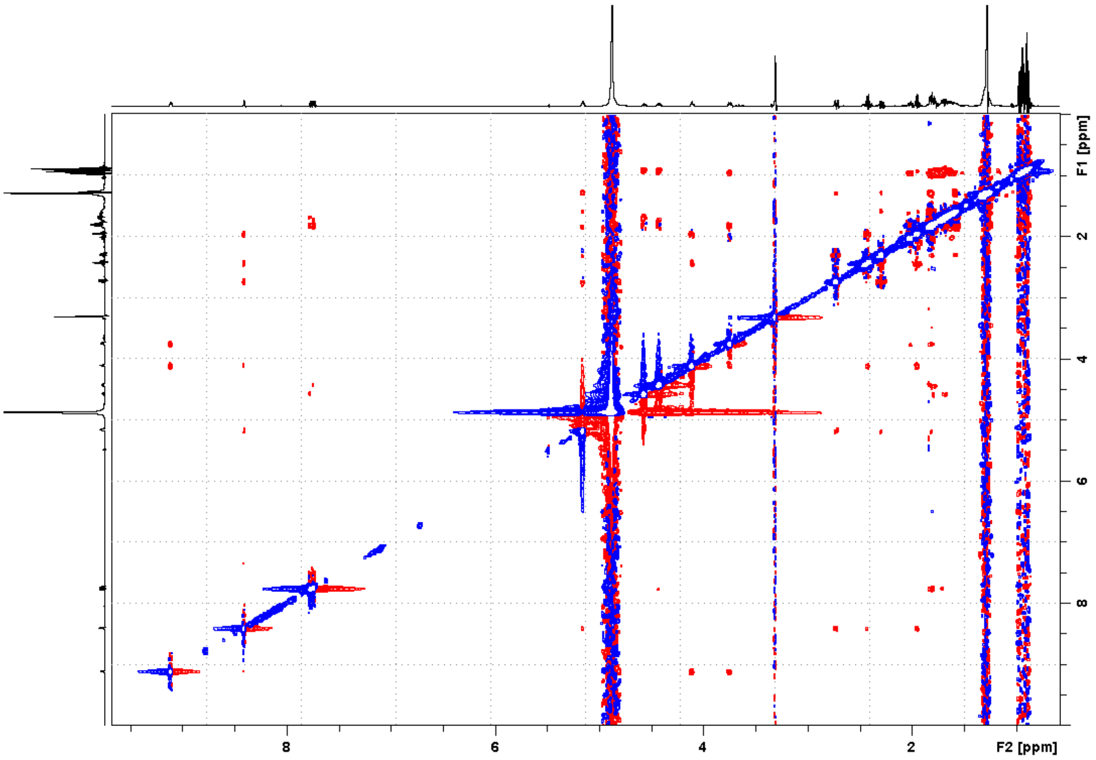

Figure S7. NOESY spectrum of compound **1** in CD_3OH.

Range : 600-820 m/z

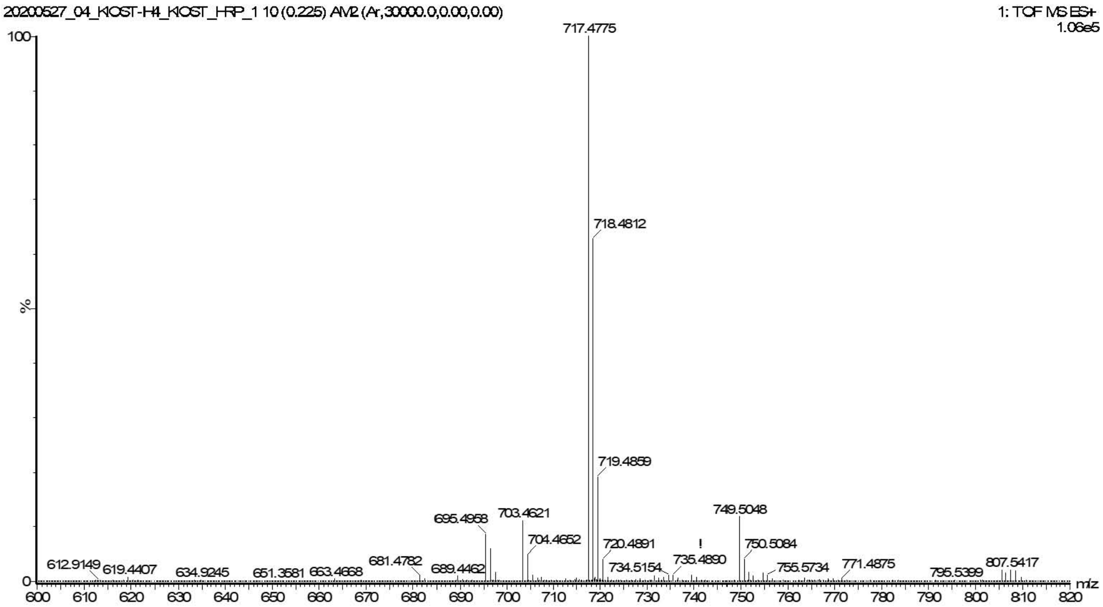

Elemental Composition Report

Single Mass Analysis
Tolerance = 5.0 PPM / DBE: min = -1.5, max = 100.0
Element prediction: Off
Number of isotope peaks used for i-FIT = 3

Monoisotopic Mass, Even Electron Ions
Elements Used:
C: 0-38 H: 0-70 N: 0-4 O: 0-8 Na: 0-1

Minimum:				-1.5				
Maximum:		100.0	5.0	100.0				
Mass	Calc. Mass	mDa	PPM	DBE	i-FIT	Norm	Conf(%)	Formula
695.4958	695.4959	-0.1	-0.1	6.5	267.1	0.117	88.94	C37 H67 N4 O8
	695.4935	2.3	3.3	3.5	269.2	2.202	11.06	C35 H68 N4 O8 Na
717.4775	717.4778	-0.3	-0.4	6.5	465.6	n/a	n/a	C37 H66 N4 O8 Na

Figure S8. HR-ESIMS spectrum of compound **1**.

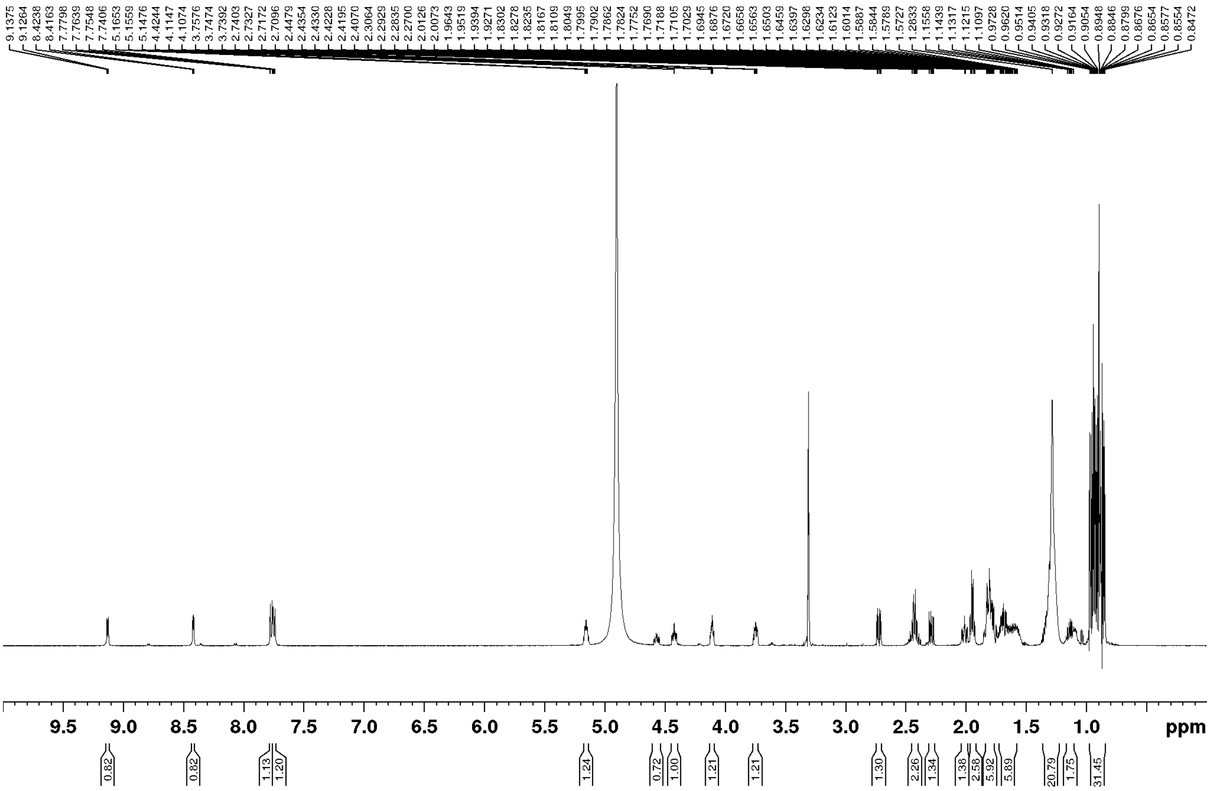

Figure S9. ^{1}H NMR spectrum of compound **2** (600 MHz, CD_3OH).

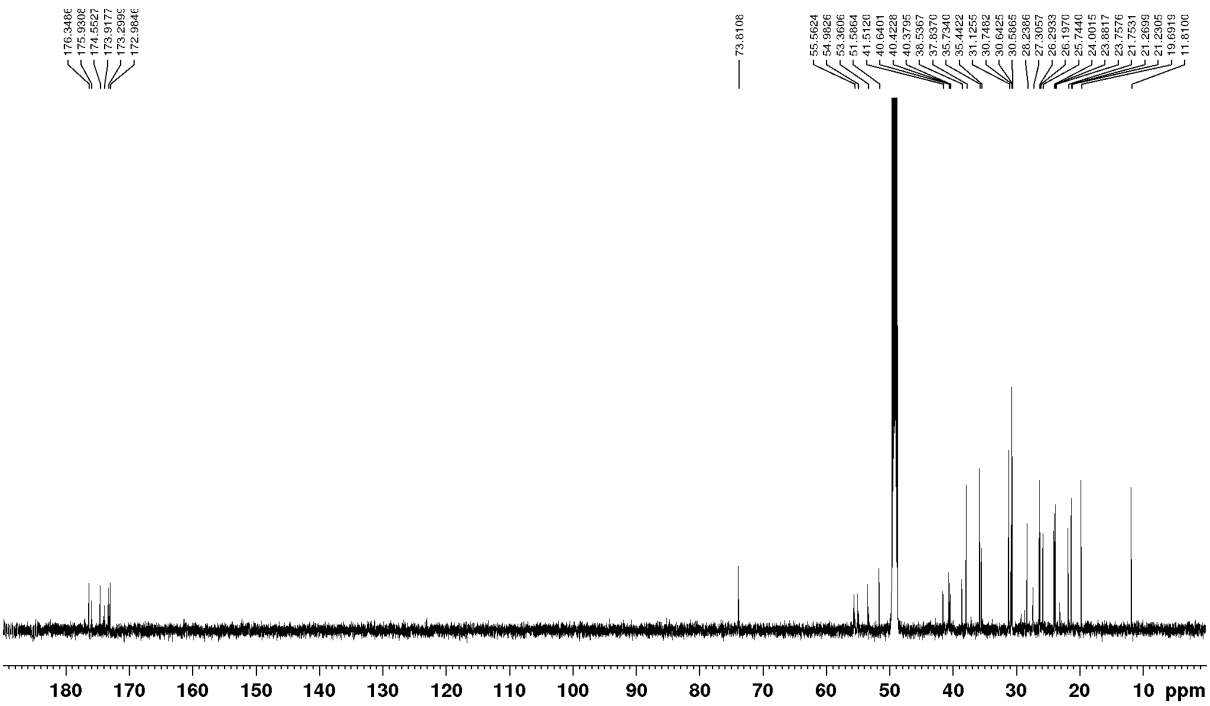

Figure S10. ^{13}C NMR spectrum of compound **2** (150 MHz, CD_3OH).

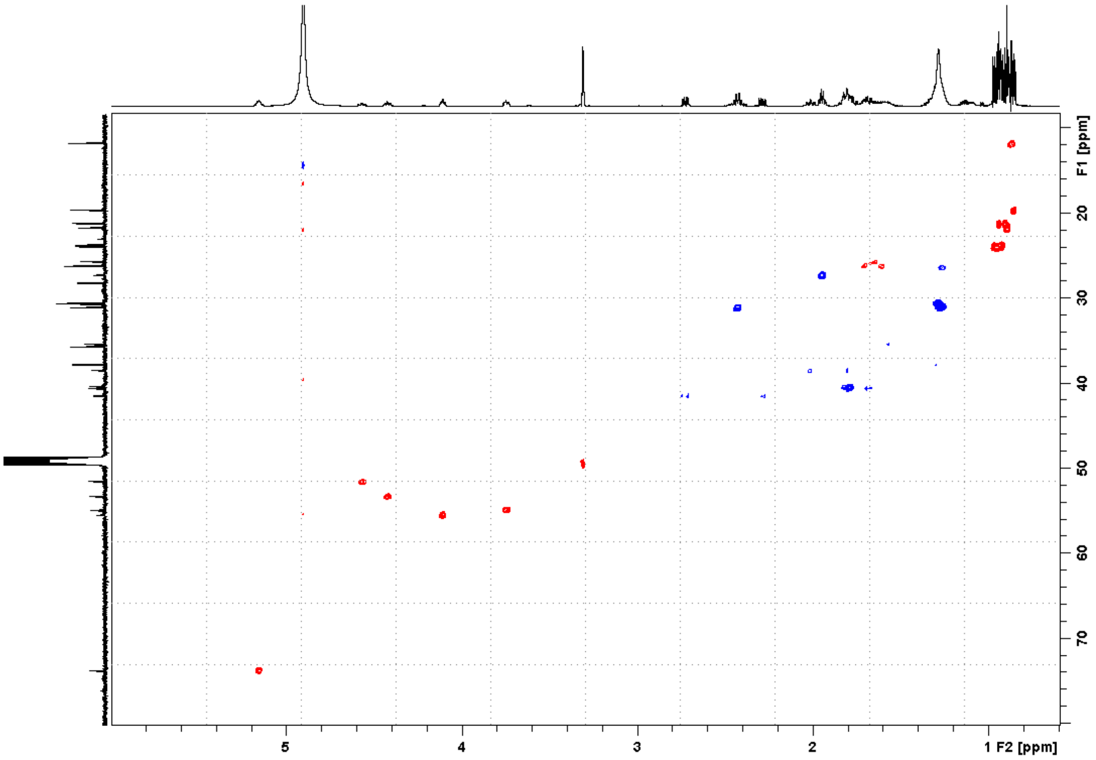

Figure S11. HSQC spectrum of compound **2** in CD_3OH.

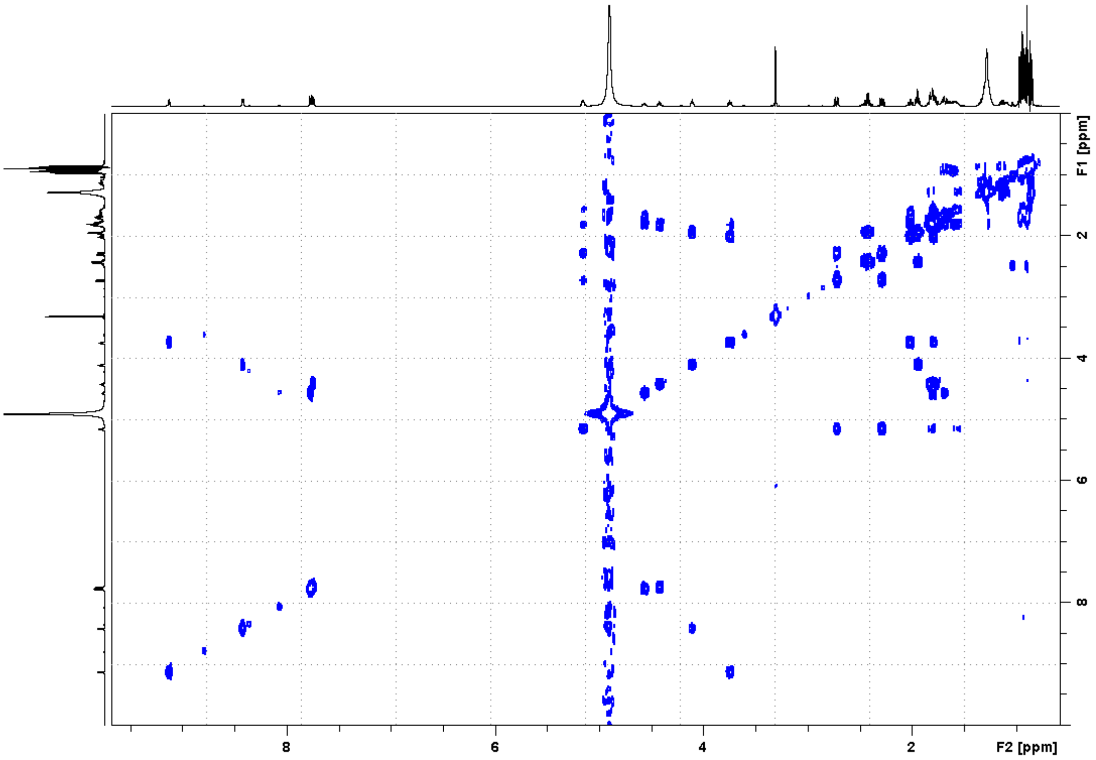

Figure S12. COSY spectrum of compound **2** in CD_3OH.

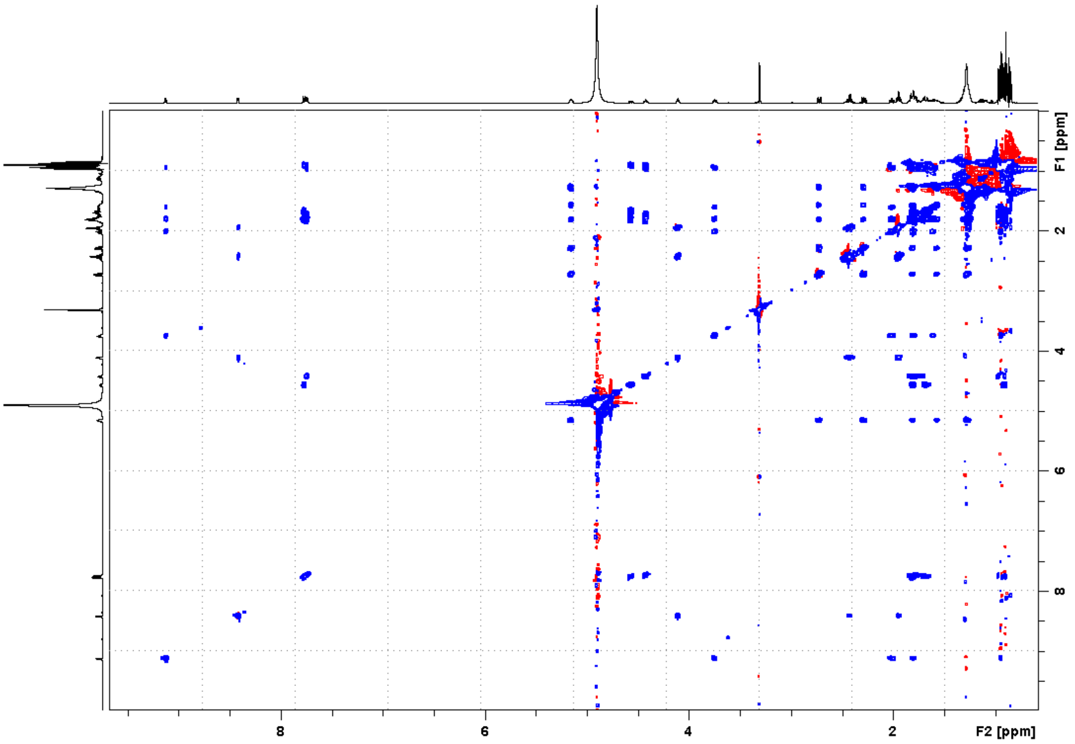

Figure S13. TOCSY spectrum of compound **2** in CD_3OH.

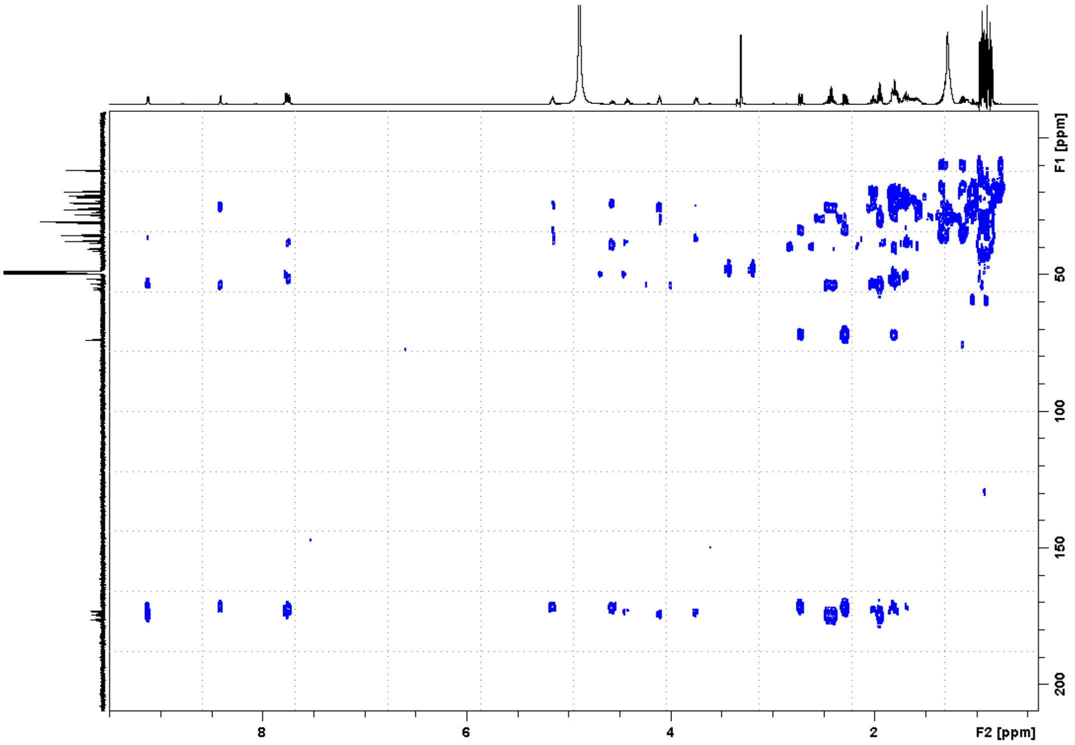

Figure S14. HMBC spectrum of compound **2** in CD_3OH.

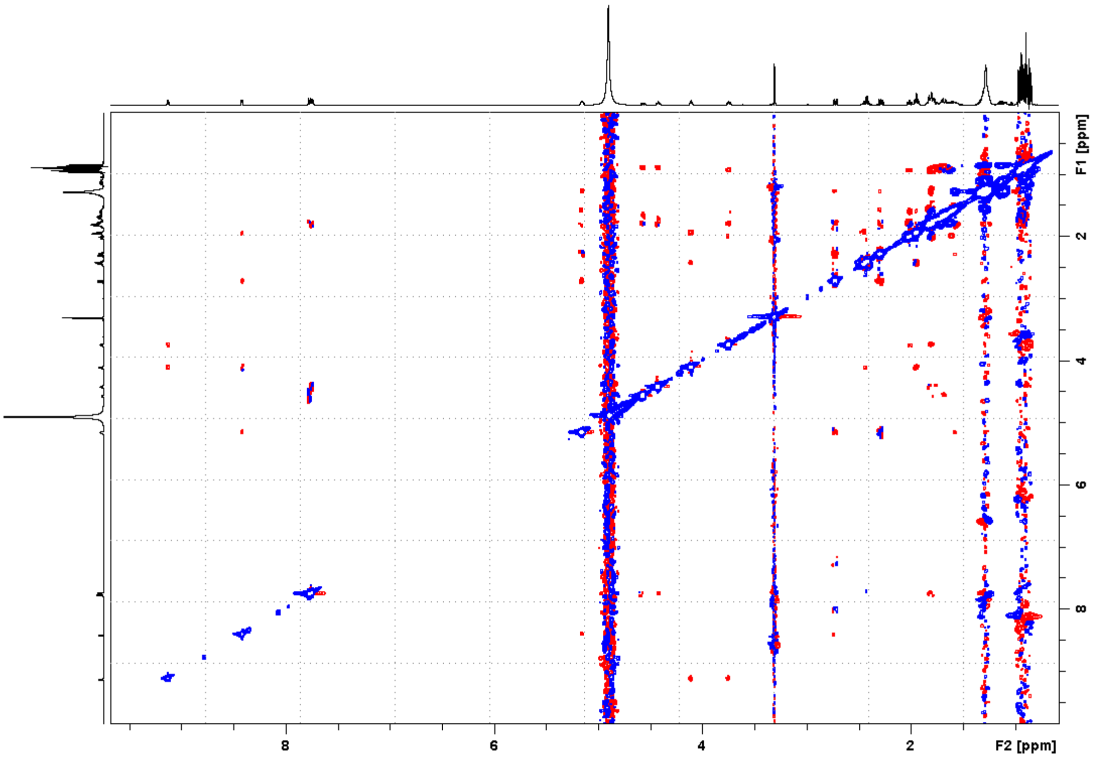

Figure S15. ROESY spectrum of compound **2** in CD_3OH.

Sample : 03

(+) ESI-MS

Range : 50-1200 m/z

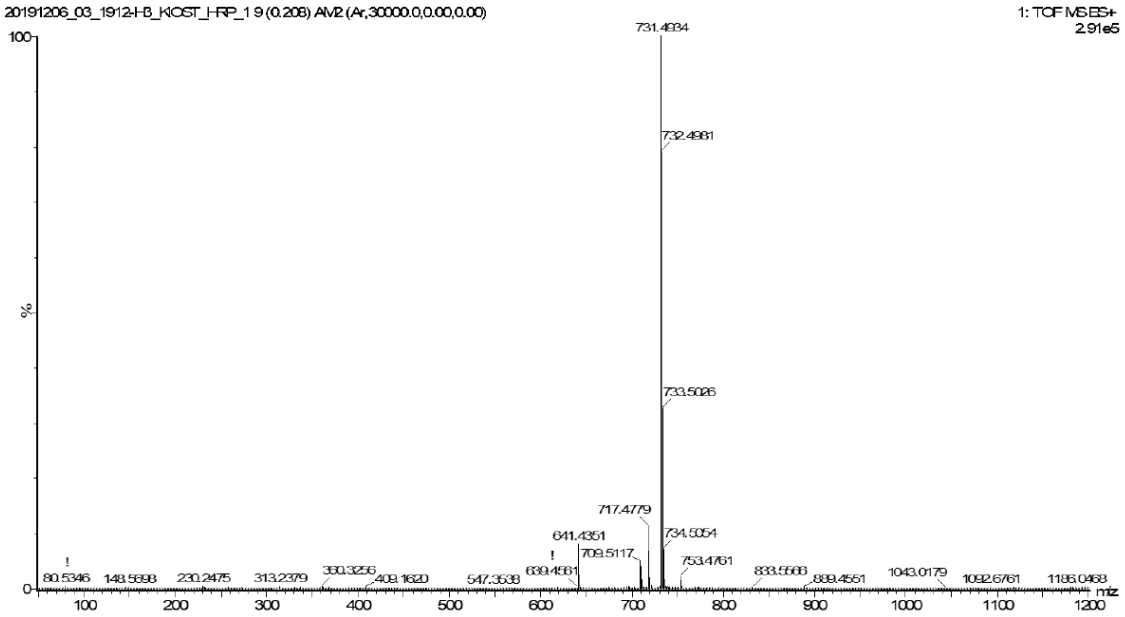

Elemental Composition Report

Single Mass Analysis
Tolerance = 5.0 PPM / DBE: min = -1.5, max = 100.0
Element prediction: Off
Number of isotope peaks used for i-FIT = 3

Monoisotopic Mass, Even Electron Ions
24 formula(e) evaluated with 2 results within limits (up to 100 closest results for each mass)
Elements Used:
C: 1-50 H: 1-70 N: 4-4 O: 8-9 Na: 0-1

Minimum:				-1.5				
Maximum:		100.0	5.0	100.0				
Mass	Calc. Mass	mDa	PPM	DBE	i-FIT	Norm	Conf(%)	Formula
731.4934	731.4935	-0.1	-0.1	6.5	638.4	0.898	40.74	C38 H68 N4 O8 Na
	731.4959	-2.5	-3.4	9.5	638.1	0.523	59.26	C40 H67 N4 O8

Figure S16. HR-ESIMS spectrum of compound **2**.

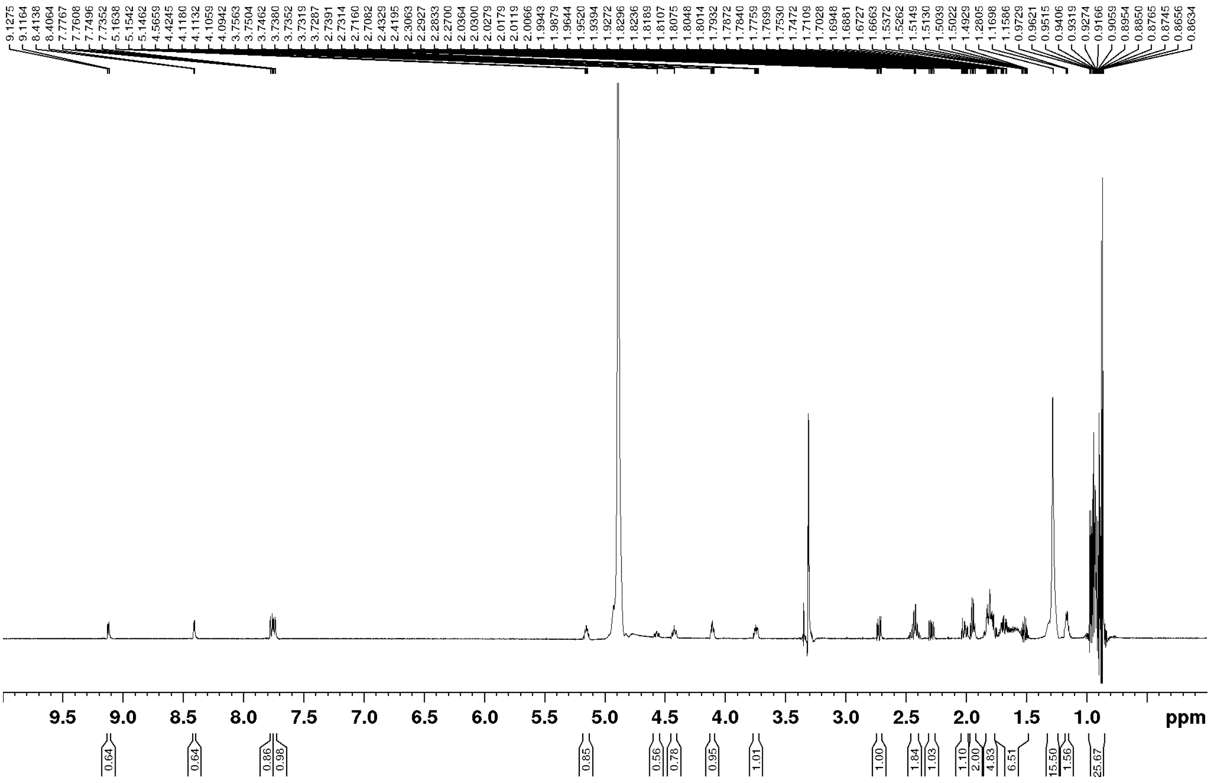

Figure S17. ^{1}H NMR spectrum of compound **3** (600 MHz, CD_3OH).

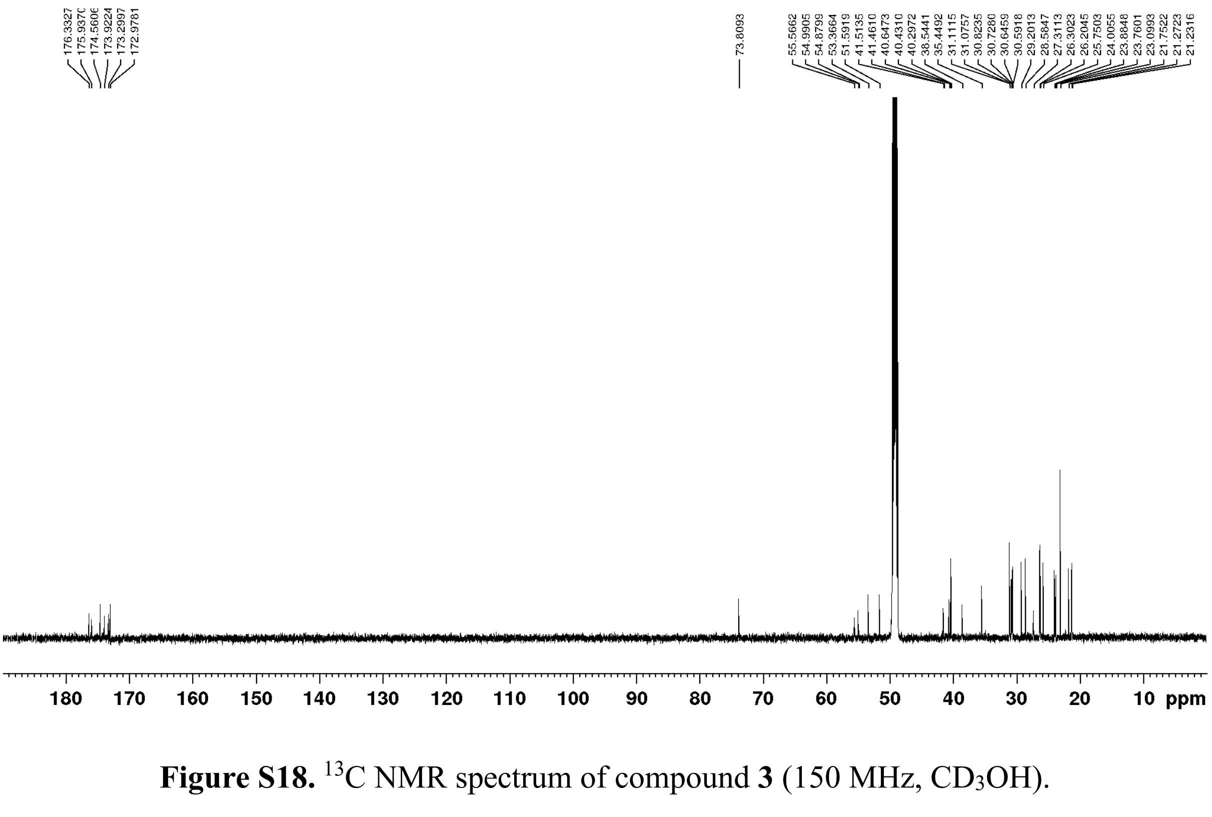

Figure S18. ^{13}C NMR spectrum of compound **3** (150 MHz, CD_3OH).

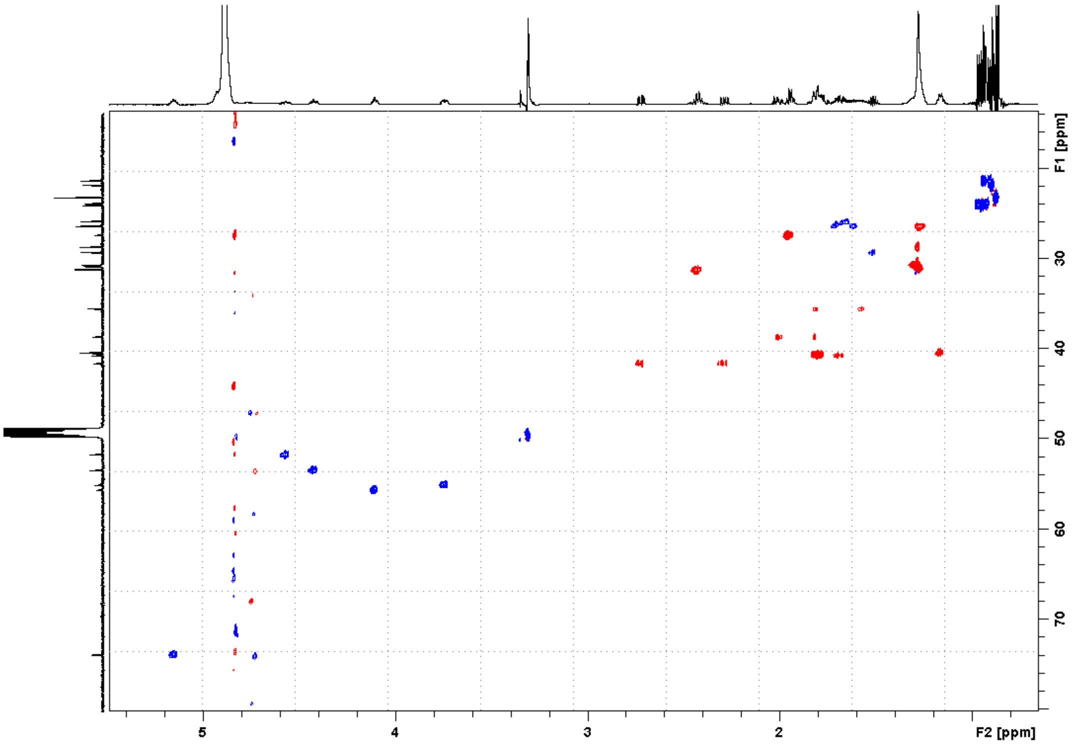

Figure S19. HSQC spectrum of compound **3** in CD_3OH.

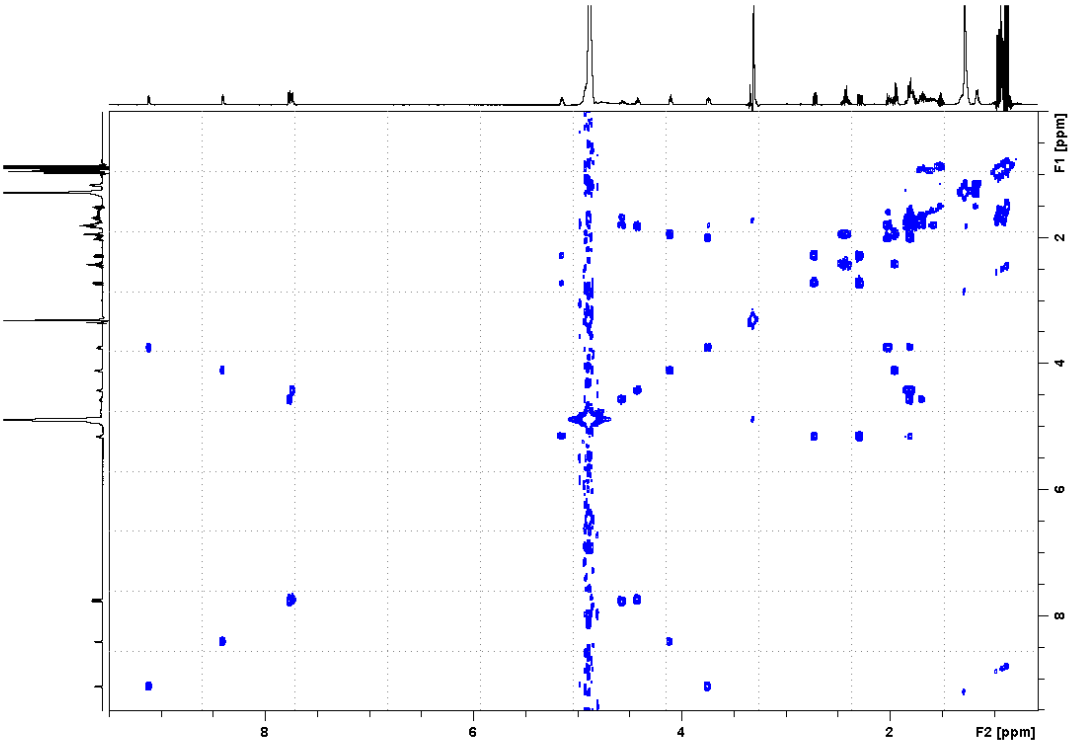

Figure S20. COSY spectrum of compound **3** in CD_3OH.

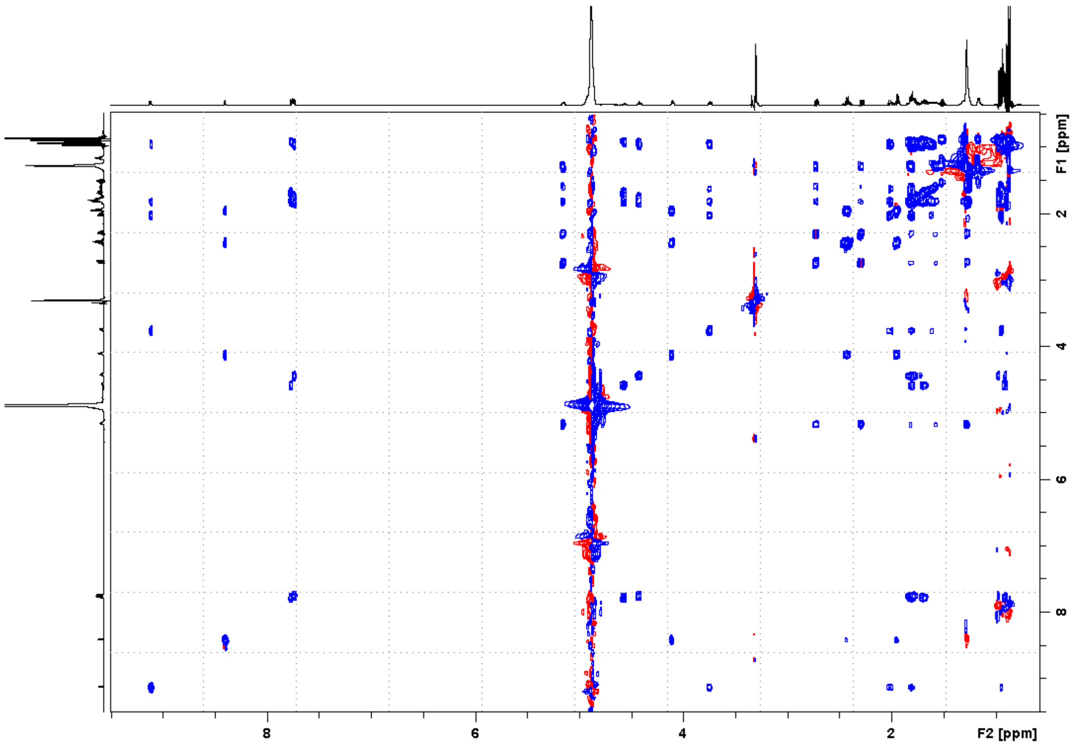

Figure S21. TOCSY spectrum of compound **3** in CD_3OH.

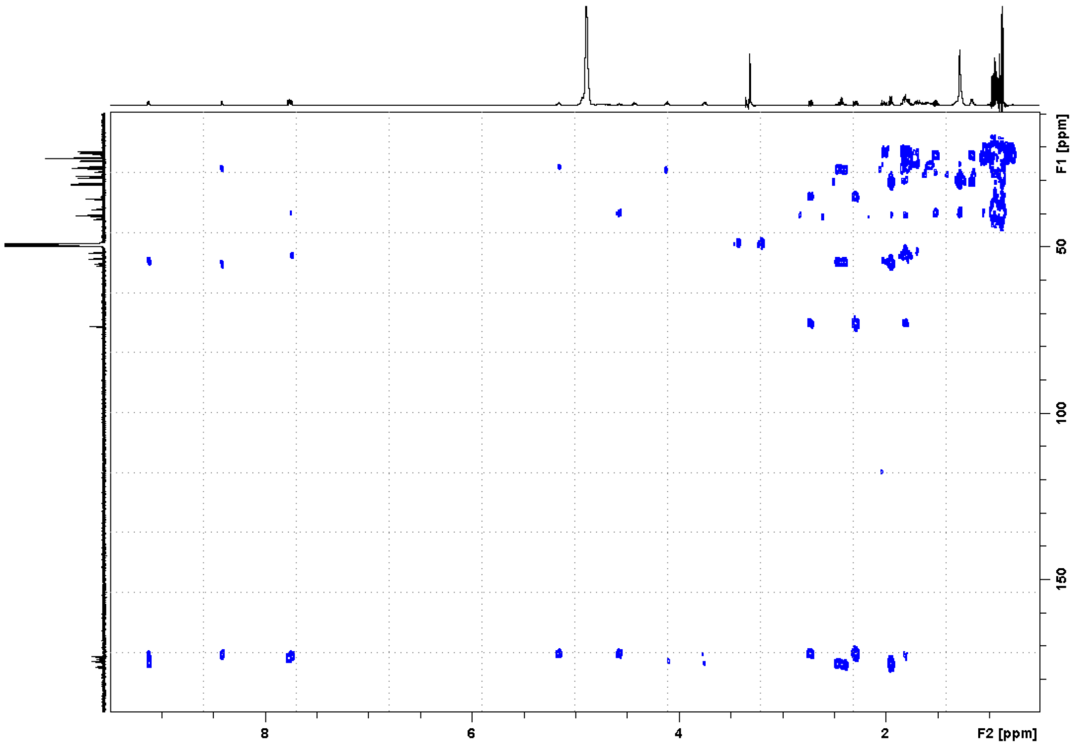

Figure S22. HMBC spectrum of compound **3** in CD_3OH.

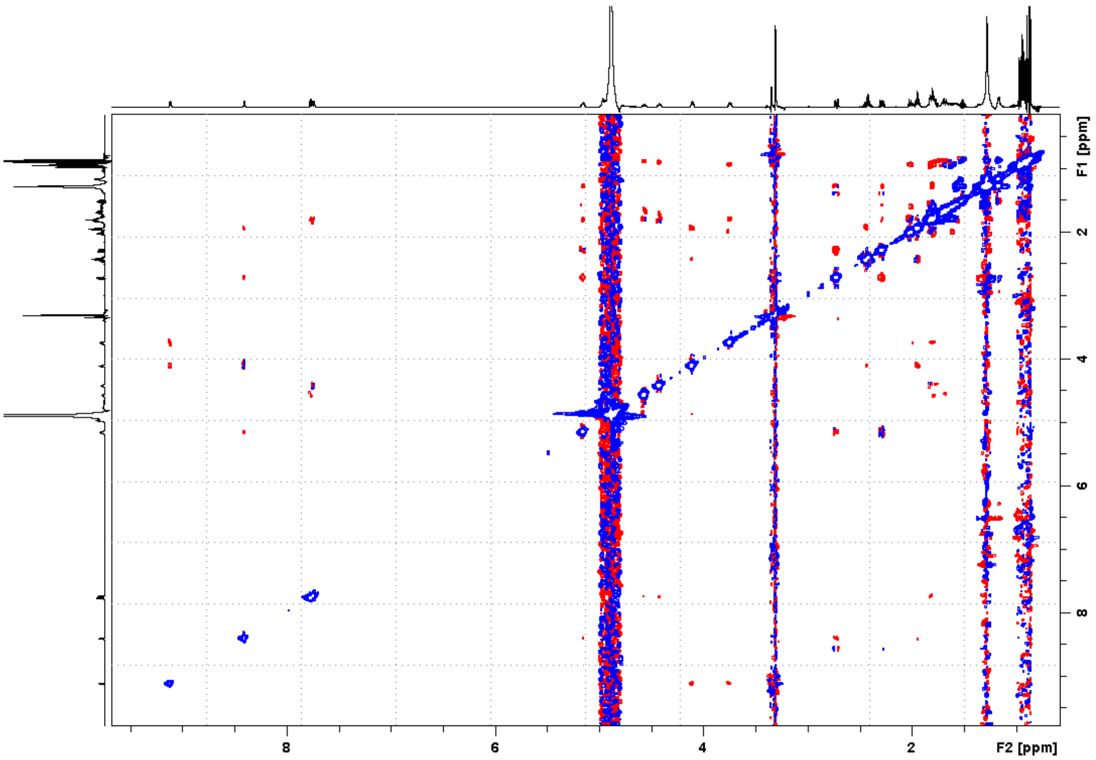

Figure S23. ROESY spectrum of compound **3** in CD_3OH.

Sample : 04
(+) ESI-MS
Range : 50-1200 m/z

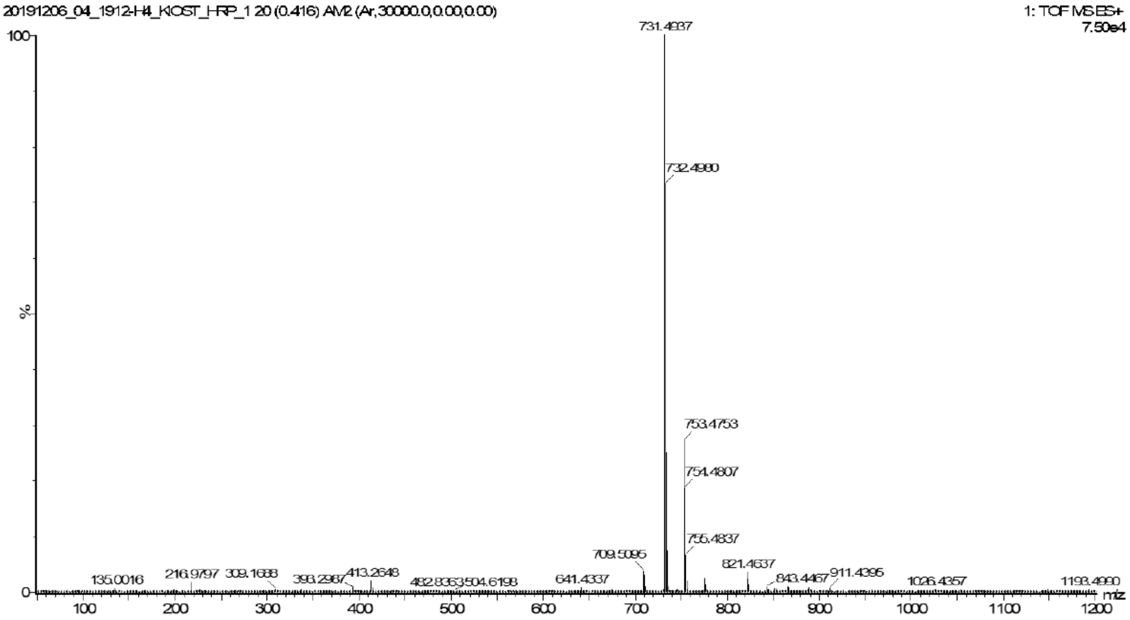

Elemental Composition Report

Single Mass Analysis
Tolerance = 5.0 PPM / DBE: min = -1.5, max = 100.0
Element prediction: Off
Number of isotope peaks used for i-FIT = 3

Monoisotopic Mass, Even Electron Ions
24 formula(e) evaluated with 2 results within limits (up to 100 closest results for each mass)
Elements Used:
C: 1-50 H: 1-70 N: 4-4 O: 8-9 Na: 0-1

Minimum:				-1.5				
Maximum:		100.0	5.0	100.0				
Mass	Calc. Mass	mDa	PPM	DBE	i-FIT	Norm	Conf(%)	Formula
731.4937	731.4935	0.2	0.3	6.5	511.5	0.833	43.48	C38 H68 N4 O8 Na
	731.4959	-2.2	-3.0	9.5	511.3	0.571	56.52	C40 H67 N4 O8

Figure S24. HR-ESIMS spectrum of compound **3**.

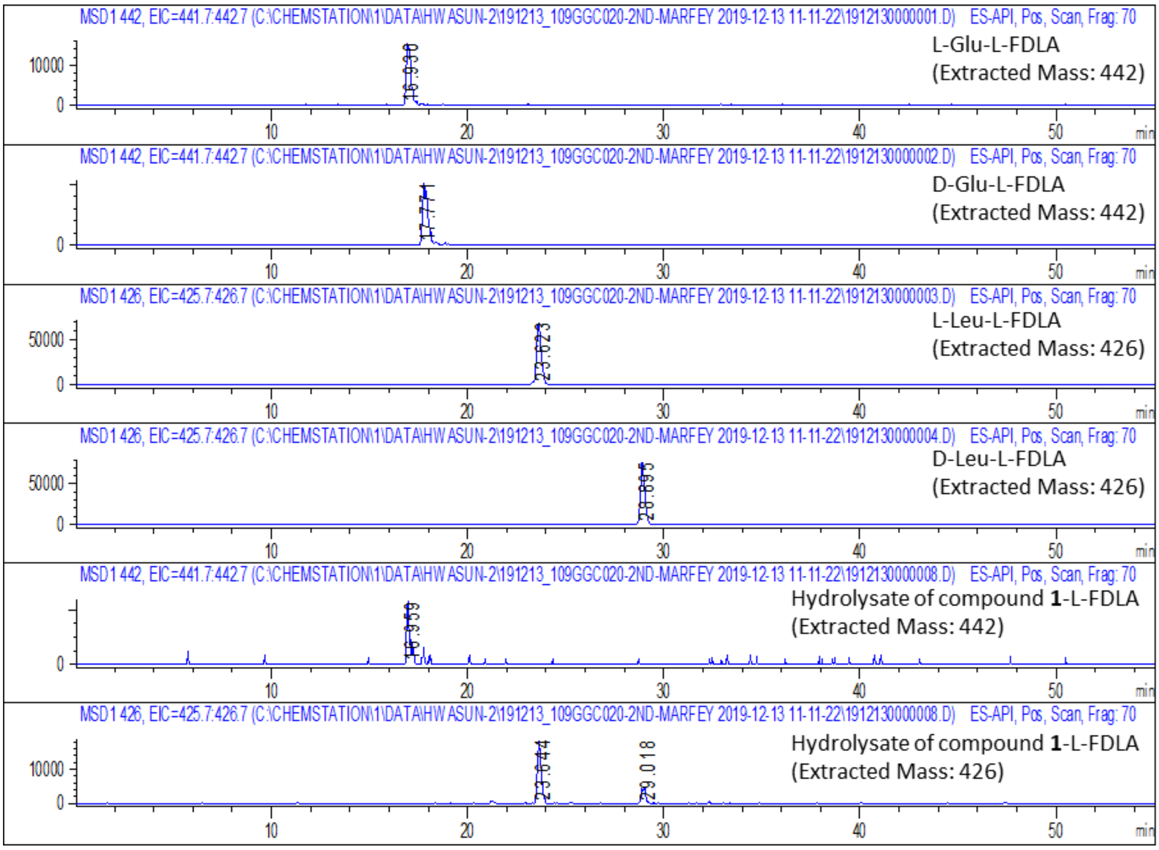

Extract ion mode, isolation condition: column: YMC ODS-A, 250 × 4.6 mm, 5 μm; flow rate: 0.5 mL/min; UV: 340 nm; gradient MeCN-H_2O (+0.02% TFA) solvent system (40% MeCN for 5 min, 40–80% MeCN over 20 min and 80% MeCN

Figure S25. HPLC analysis of amino acids in **1** through the total hydrolysis.

Table S1. Retention times (t_R, min) of L-FDLA derivatives of the hydrolysate of **1**

Amino acids	Detected *m/z*	Hydrolysate of **1**	t_R of standards
L-Glu	442	16.9	16.9
D-Glu	442	-	17.8
L-Leu	426	23.6	23.6
D-Leu	426	29.0	28.9

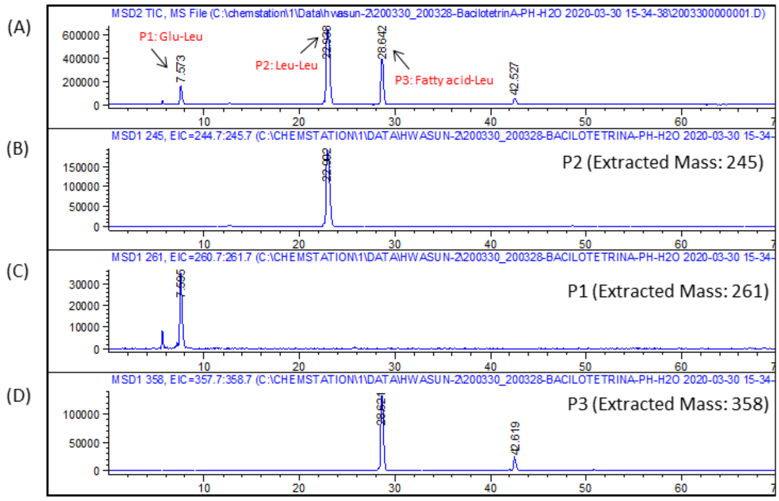

(A) SIM mode: *m/z* 245, 261 and 358 (B) extracted mass (*m/z* 245) of P2 (C) extracted mass (*m/z* 261) of P1 (D) extracted mass (*m/z* 358) of P3. Isolation condition: column: YMC ODS-A, 250 × 4.6 mm, 5 μm; flow rate: 0.5 mL/min; UV: 224 nm; gradient MeCN-H_2O (+0.02% TFA) solvent system (20% MeCN for 10 min, 20–100% MeCN over 40 min and 100% MeCN for 10 min);

Figure S26. HPLC-VWD-MS chromatogram of the partial hydrolysates of **1**.

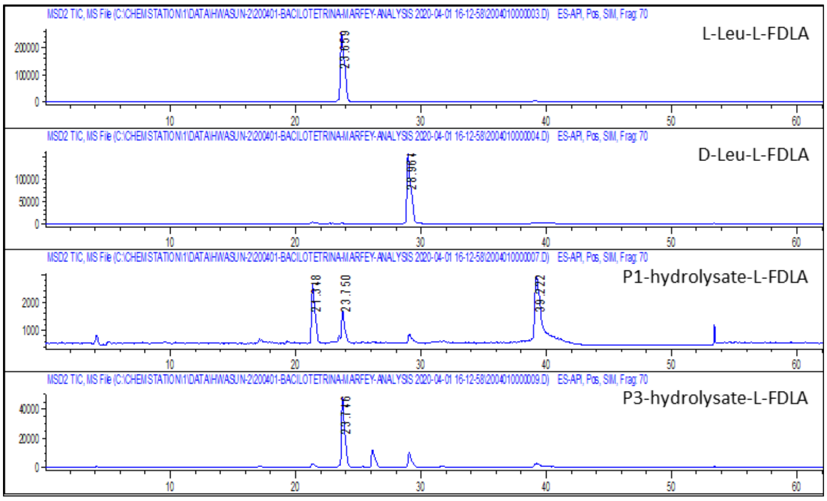

SIM mode: *m/z* 426, Column: YMC ODS-A, 250 × 4.6 mm, 5 μm; flow rate: 0.5 mL/min; UV: 340 nm; gradient MeCN-H_2O (+0.02% TFA) solvent system (40% MeCN for 5 min, 40–80% MeCN over 20 min and 80% MeCN for 5 min)

Figure S27. HPLC analysis of L-FDLA derivatives of P1 and P3 hydrolysates.

Table S2. Retention times (t_R, min) of L-FDLA derivatives of P1 and P3 hydrolysates

Amino acids	Detected *m/z*	P1 Hydrolysate	P3 Hydrolysate	t_R of standards
L-Leu	426	23.7	23.7	23.6
D-Leu	426	-	-	29.0

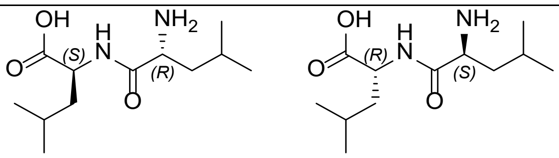

Figure S28. Structures of standard dipeptides.

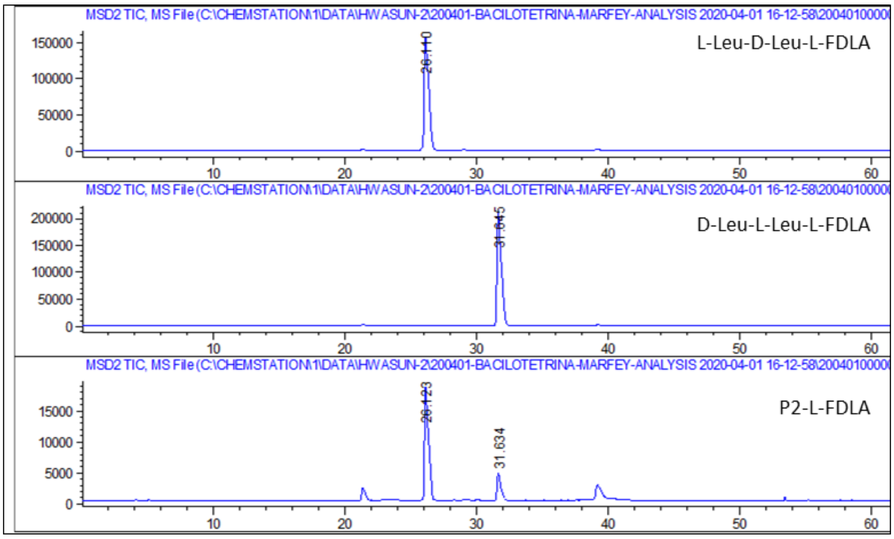

SIM mode: *m/z* 589, Column: YMC ODS-A, 250 × 4.6 mm, 5 μm; flow rate: 0.5 mL/min; UV: 340 nm; gradient MeCN-H_2O (+0.02% TFA) solvent system (40% MeCN for 5 min, 40–80% MeCN over 20 min and 80% MeCN for 5 min)

Figure S29. HPLC analysis of L-FDLA derivatives of P2.

Table S3. Retention times (t_R, min) of L-FDLA derivatives of P2

Amino acids	Detected *m/z*	P3	t_R of standards
L-Leu-D-Leu	589	26.1	26.1
D-Leu-L-Leu	589	31.6	31.6

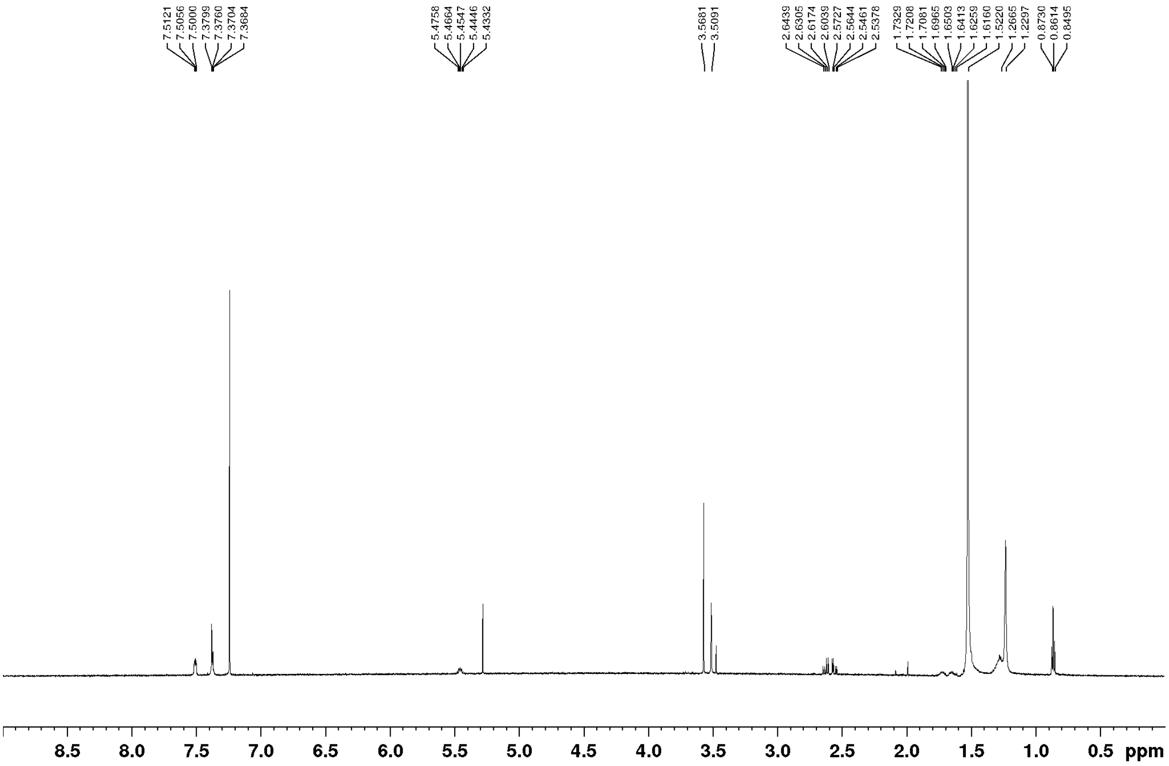

Figure S30. ^{1}H NMR spectrum of the (*S*)-MTPA ester (**1b**) (600 MHz, $CDCl_3$).

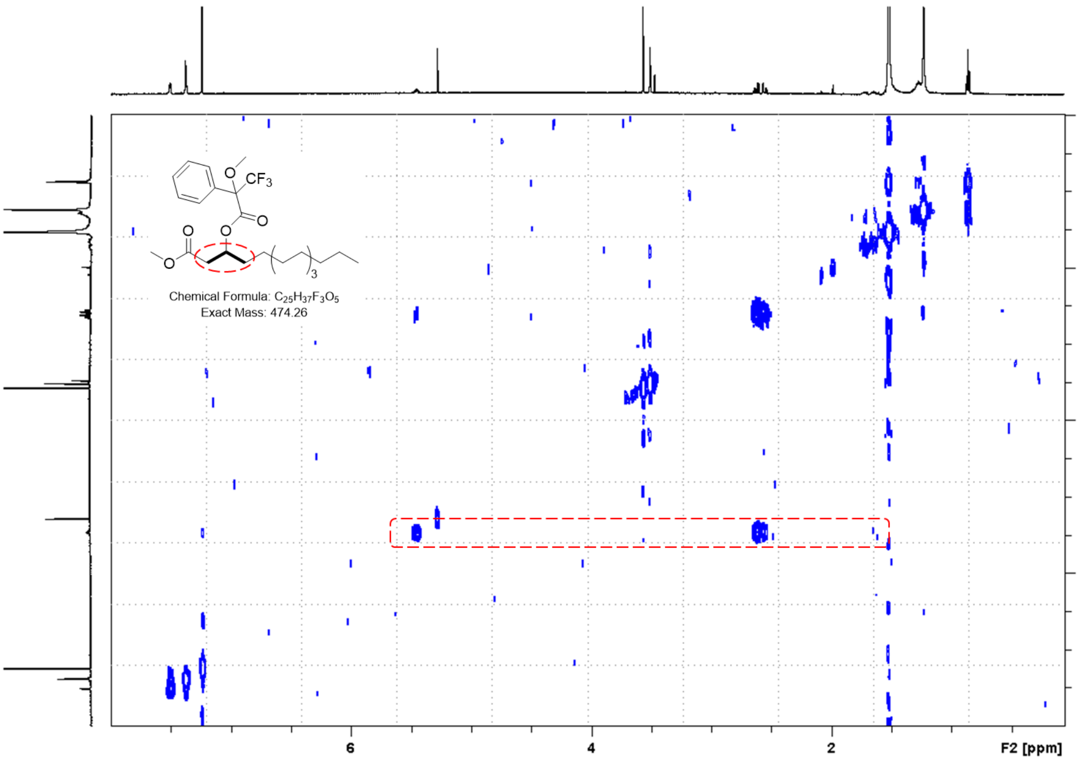

Figure S31. COSY spectrum of the (*S*)-MTPA ester (**1b**) in $CDCl_3$.

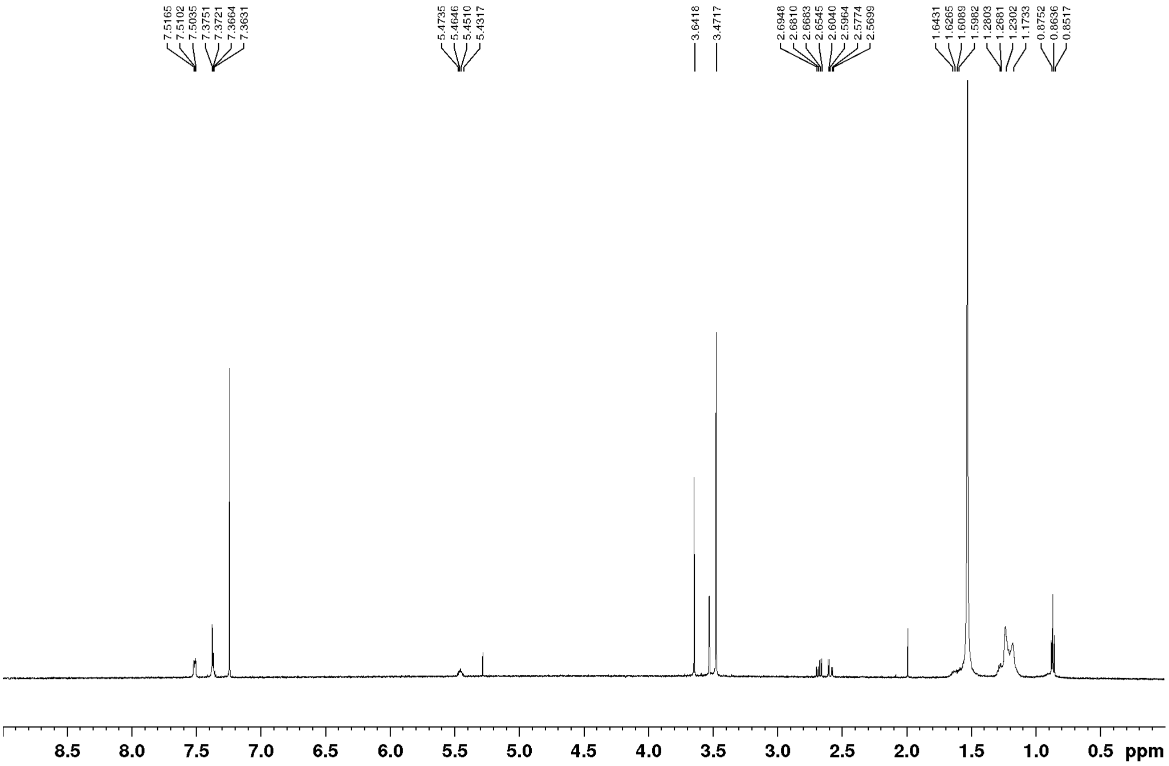

Figure S32. ^{1}H NMR spectrum of the (*R*)-MTPA ester (**1c**) (600 MHz, $CDCl_3$).

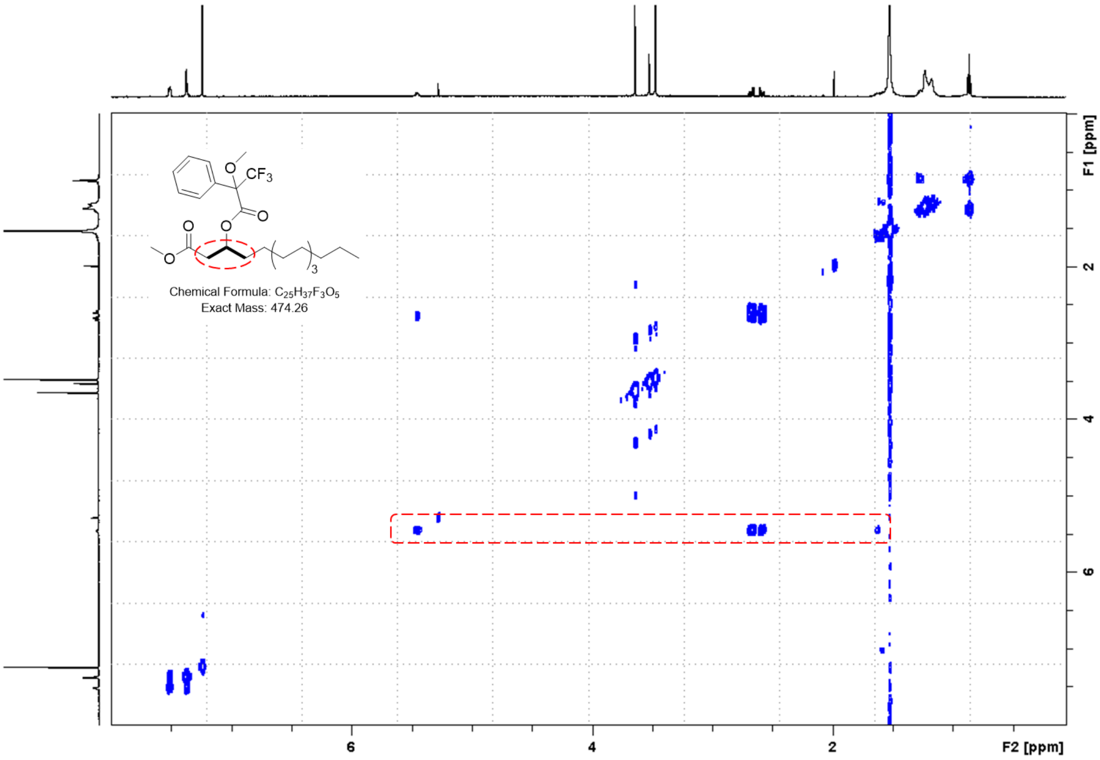

Figure S33. COSY spectrum of the (*R*)-MTPA ester (**1c**) in $CDCl_3$.

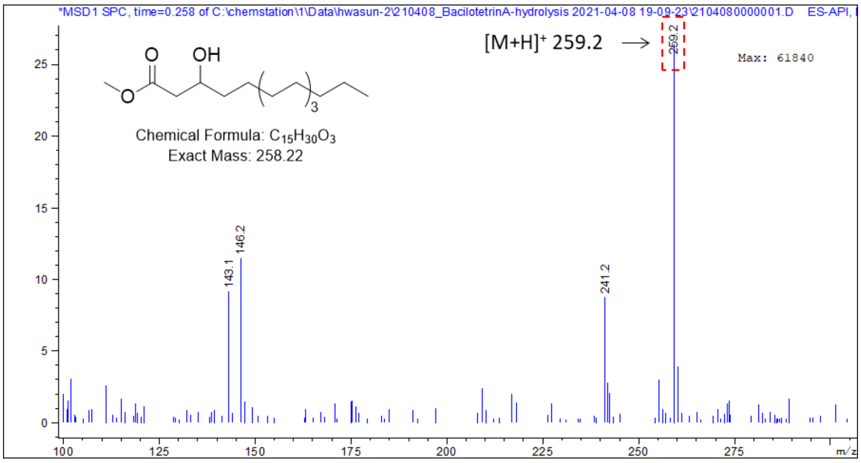

Figure S34. LR-EIMS spectrum of **1a**.

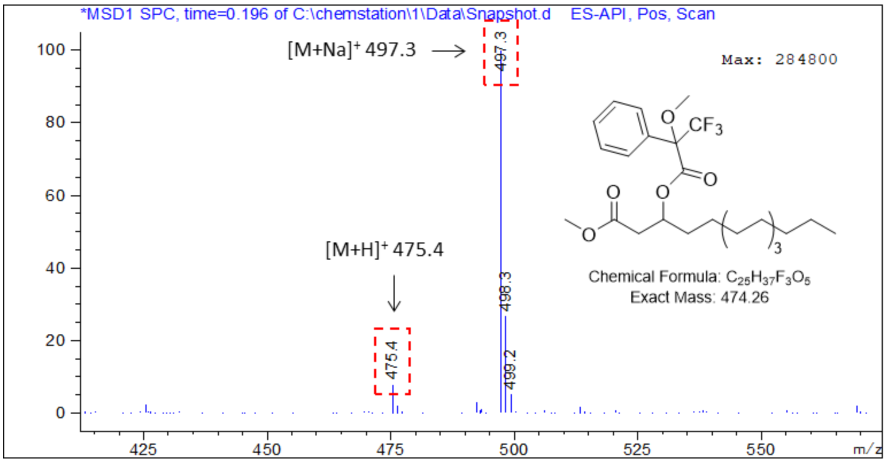

Figure S35. LR-EIMS spectrum of the (*S*)-MTPA ester (**1b**).

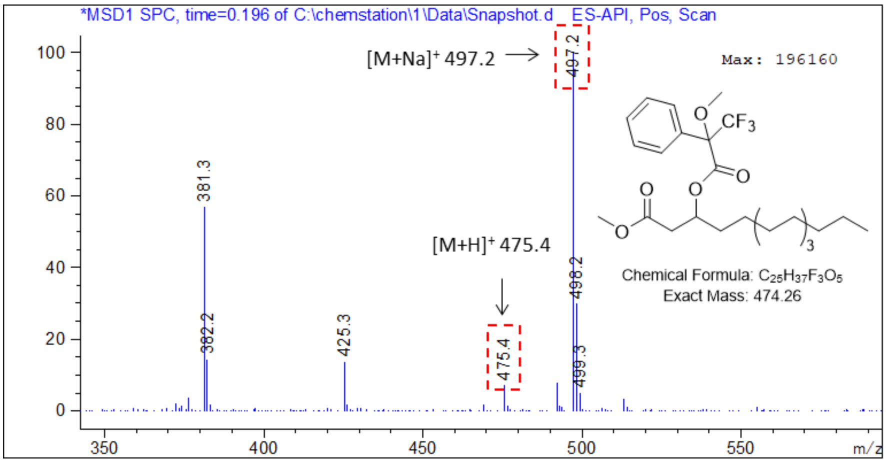

Figure S36. LR-EIMS spectrum of the (*R*)-MTPA ester (**1c**).

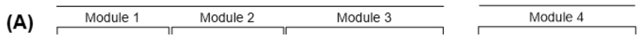

Koglin, A.; Lohr, F.; Bernhard, F.; Rogov, V. V.; Frueh, D. P.; Strieter, E. R.; Mofid, M. R.; Guntert, P.; Wagner, G.; Walsh, C. T.; Marahiel, M. A.; Dotsch, V., Structural basis for the selectivity of the external thioesterase of the surfactin synthetase. *Nature* **2008**, 454, (7206), 907-911.

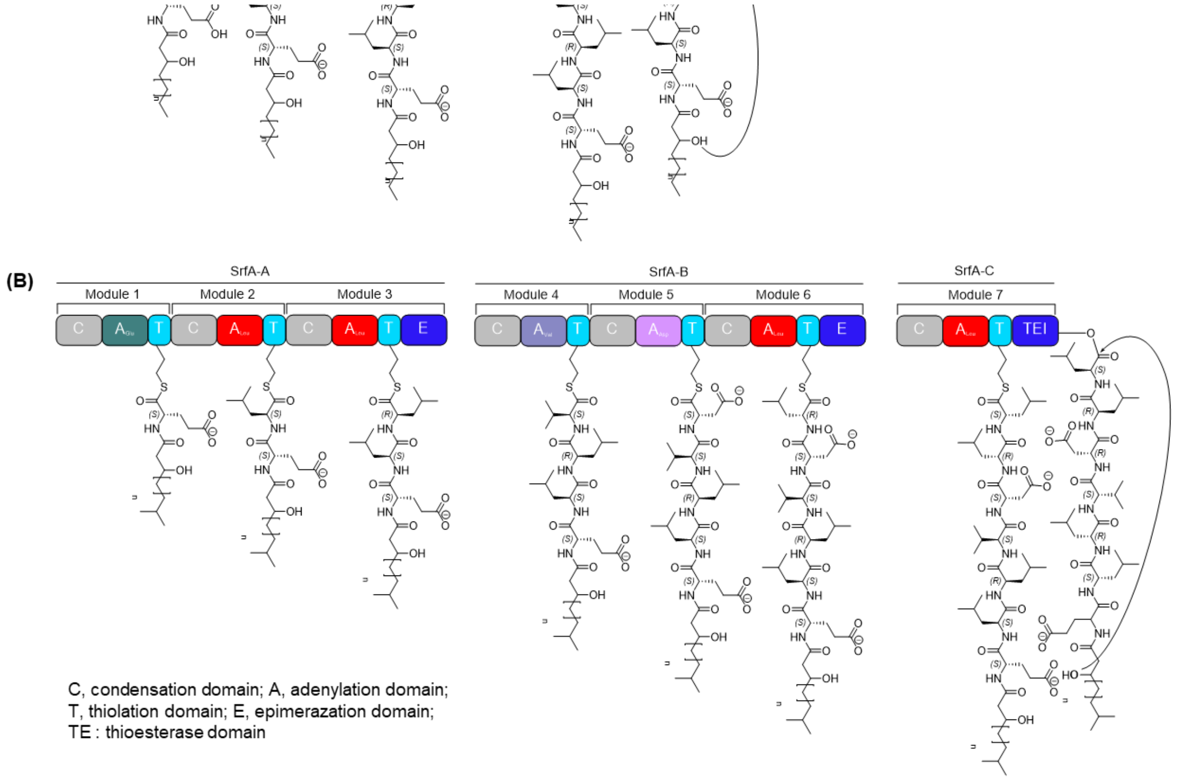

Figure S37. (A) Plausible biosynthetic pathway of compounds **1–3**; (B) biosynthetic pathway of surfactin.

Table S4. ^{1}H and ^{13}C NMR data for data for reported and revised bacilotetrin A (**4**) (500 MHz, CD_3OD)

		Reported bacilotetrin A (**4**)		Revised bacilotetrin A (**4**)	
Position		δ_C, type	δ_H, m (*J* in Hz)	δ_C, type	δ_H, m (*J* in Hz)
Glu					
	1	173.0, C		175.9, C	
	2	51.6, CH	4.58, m	55.6, CH	4.10, m
	3	35.5, CH_2	1.81, m	27.4, CH_2	1.94, q (7.4)
	4	31.5, CH_2	2.43, m	31.5, CH_2	2.42, m
	5	173.3, C		176.0, C	
	NH		7.33, d (8.5)[a]		8.42, d (4.5)
Leu-1					
	1	175.9, C		173.9, C	
	2	55.5, CH	4.11, t (7.5)	54.9, CH	3.75, m
	3	40.7, CH_2	1.65 m	38.5, CH_2	2.01, ddd (14.6, 11.0, 3.8)
					1.80, o.l[c]
	4	26.4, CH	1.68, m	26.28, CH	1.60, o.l[c]
	5	21.8, CH_3	0.91, m	21.2, CH_3	0.93, o.l[c]
	6	24.1, CH_3	0.95, m	24.0, CH_3	0.94, o.l[c]
	NH		8.40, d (4.5)[a]		9.11, d (6.7)
Leu-2					
	1	173.3, C		174.5, C	
	2	54.9, CH	3.75, dd (11.0, 4.0)	53.3, CH	4.42, ddd (12.1, 8.6, 3.6)
	3	38.6, CH_2	1.65, m	40.4, CH_2	1.80, o.l[c]
	4	26.4 CH	1.68, m	26.26, CH	1.70, o.l[c]
	5	21.3, CH_3	0.91, m	21.3, CH_3	0.89, o.l[c]
	6	23.9, CH_3	0.95, m	23.9, CH_3	0.96, o.l[c]
	NH		9.10, d (6.5)[b]		7.74, d (8.6)
Leu-3					
	1	174.6, C		172.9, C	
	2	53.4, CH	4.44, m	51.5, CH	4.57, m
	3	40.5, CH_2	1.65, m	40.5, CH_2	1.80, o.l[c]
					1.69, o.l[c]
	4	25.8, CH	1.65, m	25.72, CH	1.64, o.l[c]
	5	21.3, CH_3	0.91, m	21.8, CH_3	0.91, o.l[c]
	6	23.9, CH_3	0.95, m	23.8, CH_3	0.92, o.l[c]
	NH		7.43, d (9.5)[b]		7.77, d (9.6)
β-OH acid					
	1	173.0, C		173.2, C	
	2	41.6, CH_2	2.73, dd (13.5, 4.5)	41.5, CH_2	2.72, dd (13.8, 4.6)
			2.29, dd (14.0, 8.5)		2.29, dd (13.8, 8.1)
	3	73.8, CH	5.16, m	73.8, CH	5.16, tt (7.8, 5.3)
	4	40.4, CH_2	1.58, m	35.5, CH_2	1.80, o.l[c]
					1.57, o.l[c]

Table S4. *Cont.*

	Reported bacilotetrin A (**4**)		Revised bacilotetrin A (**4**)	
Position	δ_C, type	δ_H, m (J in Hz)	δ_C, type	δ_H, m (J in Hz)
β-OH acid				
5				
6				
7	26.17 – 30.8, CH_2	1.29, brs		
8			26.17 – 30.8, CH_2	1.28, o.l[c]
9				
10				
11	30.6, CH	1.50, m		
12	33.2, CH_2	1.29, brs	33.1, CH_2	1.29, o.l[c]
13	14.6, CH_3	0.89, m	23.8, CH_2	1.33, o.l[c]
14	23.9, CH_3	0.90, m	14.5, CH_3	0.89, o.l[c]

[a,b]Chemical shifts and coupling constants were determined in CD3OH and DMSO, respectively. [c]Signals were overlapped with other signals.
